# Apocynaceae wood evolution matches key morphological innovations

**DOI:** 10.1002/ajb2.16436

**Published:** 2024-11-22

**Authors:** Vicky Beckers, Mary Endress, Pieter Baas, Erik Smets, Frederic Lens

**Affiliations:** ^1^ Naturalis Biodiversity Center Darwinweg 2 Leiden 2333CR The Netherlands; ^2^ Plant Sciences, Institute of Biology Leiden Leiden University Sylviusweg 72 Leiden 2333BE The Netherlands; ^3^ Department of Systematic and Evolutionary Botany University of Zurich Zollikerstrasse 107 Zurich 8008 Switzerland; ^4^ Ecology, Evolution and Biodiversity Conservation, KU Leuven Kasteelpark Arenberg 31, Box 2435 Leuven 3001 Belgium

**Keywords:** evolution, lianas, morphology, phylogenetic comparative methods, synnovations, wood anatomy

## Abstract

**Premise:**

This paper provides an overview of the wood anatomy of the dogbane family (Apocynaceae), reconstructs wood anatomical trait evolution, and links this evolution with woody growth‐form transitions and floral and seed trait innovations across the family.

**Methods:**

Over 200 published wood anatomical descriptions were revised, and original light microscopic sections were made and described for another 50 species. Changes in wood anatomical characters through time were visualized with ancestral state reconstructions. Tests for correlated evolution were performed using a combined data set of anatomical and key floral and seed traits to identify potential synnovations and traits associated with growth‐form adaptations.

**Results:**

There was a shift toward a suite of wood anatomical traits that separate the rauvolfioids and early‐branching apocynoids from the core apocynoids, including an increased presence of vessel multiples, vessel dimorphism, laticifers, vascular (cambial) variants, and paratracheal axial parenchyma. The presence of this trait suite, which continues in Periplocoideae, Secamonoideae, and Asclepiadoideae, coincides with a progression of floral morphological innovations that evolved on consecutive nodes in the family, and also relates to more frequent transitions toward the climbing and herbaceous habits. In addition, a considerable shortening of vessel elements and fibers along the phylogenetic backbone of the family is correlated with a general reduction in plant size.

**Conclusions:**

There are clear evolutionary transitions in the wood anatomy of Apocynaceae representing structural adaptations across the family that are associated with a quick succession of evolutionary changes of the floral bauplan.

Apocynaceae are a large pantropical plant family characterized by a great variation in growth forms, ranging from large tropical trees of 80 m to smaller trees and shrubs, lianas, succulents, and herbs, with diversity hotspots in Central and South America, Africa, and Southeast Asia (Ollerton et al., 2019). Variation in plant size follows a general reduction along the phylogenetic backbone of the family, which has resulted in a gradual reduction of the wood cylinder (Fishbein et al., 2018; Beckers et al., 2022). The family originated 86 million years ago (Ma) and underwent complex diversification patterns with high speciation rates after the Eocene climatic optimum (Bitencourt et al., 2021), which resulted in around 5350 currently recognized species, divided into five major groups (Figure 1): two paraphyletic grades (rauvolfioids and apocynoids) and three monophyletic subfamilies (Periplocoideae, Secamonoideae, and Asclepiadoideae, that were formerly considered a separate family: the Asclepiadaceae; Fishbein et al., 2018; Endress et al., 2019; Antonelli et al., 2021). These groups are characterized by differences in growth form and reproductive characters. For instance, rauvolfioids (11 tribes), the earliest‐branching group, are defined by a combination of being large trees and shrubs (Figure 1), having anthers free from the style head, a variety of seed types, and colporate pollen shed as monads (Simões et al., 2007; Endress et al., 2019). Sister to rauvolfioids is the APSA clade, which consists of the apocynoids, Periplocoideae, Secamonoideae, and Asclepiadoideae. All APSA members have anthers postgenitally attached to the style head (gynostegium; Figure 1). The apocynoids (nine tribes) are mainly characterized by a woody climbing habit (Figure 1), comose seeds, and porate pollen shed as monads (Livshultz et al., 2007). Periplocoideae range from small suffrutescent and woody shrubs to climbers and are defined by porate pollen tetrads that may be united into pollinia; these pollinia are shed onto a translator consisting of a scoop with a viscidium at the end (Figure 1). Secamonoideae have a similar woody growth form and are recognized by four pollinia per anther, which are attached to a translator functioning through a clip mechanism. Asclepiadoideae (five tribes) are by far the most species‐rich group, with over 3000 species that exhibit a broad array of growth forms, including small shrubs, woody and nonwoody climbers, succulents, and many herbaceous species. They display a variety of flower morphologies and are characterized by two pollinia per anther attached to a clip translator (Figure 1; Wanntorp, 1988; Judd et al., 1994; Fishbein, 2001; Livshultz et al., 2007).

**Figure 1:**
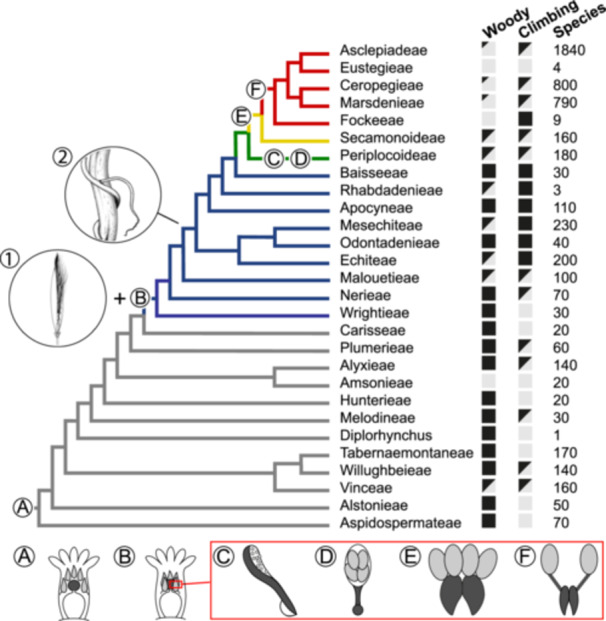
Cladogram showing phylogenetic relationships within Apocynaceae and key floral and seed morphological innovations, along with information on growth form, number of species, and evolution of the pollination system. Woody species are trees, shrubs, and/or lianas, black = presence, grey = absence. (A, B) Rauvolfioid versus APSA flower. Light gray = stamens; dark gray = style head. (A) Rauvolfioid flower with stamens free from style head; (B) APSA flower with gynostegium (anthers attached to style head). (C–F) Evolution of pollen aggregation and two morphologically distinct bauplans for translators composed of style‐head secretions and pollinaria (translator + pollinia) in Periplocoideae (C, D), Secamonoideae (E) and Asclepiadoideae (F). White = adhesive; light gray = pollen; dark gray = translator. (C) Side view of a firm scoop‐like translator with sticky adhesive lining by which the pollen (aggregated into porate tetrads) adhere to the scoop and with a sticky disc (viscidium) at the base of the translator to attach the translator to the pollinator. (D) Front view of pollinarium formed of a translator with adhesive‐lined scoop onto which porate pollen tetrads (aggregated into pollinia without a pollen wall) are shed, and with viscidium at the base. (E) Pollinarium comprising a translator with four sessile pollinia composed of inaperturate tetrads without a pollinium wall, attaching to the pollinator via a clip mechanism. (F) Pollinarium comprising a clip‐type translator and two pollinia composed of single inaperturate pollen grains with a pollinium wall and attached to the clip by arms. Additional potential drivers of diversification following Bitencourt et al. (2021): 1 = dry fruit with comose seeds, 2 = climbing habit. Tribes behind grey branches = rauvolfioids, blue = apocynoids, green = Periplocoideae, yellow = Secamonoideae, and red = Asclepiadoideae. Relationships after Fishbein et al. (2018) using the plastid phylogeny under constraint of the plastome, with updated relationships of Echiteae and sister clades based on Morales et al. (2017)—except for the position of Rhabdadenieae. Number of species per taxon and specifics on woody growth form type based on Endress et al. (2019). Botanical illustrations 1, 2, A, and B made by Esmée Winkel, C–F drawn and adapted from Endress (2003). Illustrations are not to scale.

Many of these unique reproductive Apocynaceae traits have been studied in a phylogenetic context. For instance, on the basis of aggregated pollen evolution reconstructions, pollinia were hypothesized to have evolved five times independently (three times in Periplocoideae alone [Ionta and Judd, 2007]), and there are many more trait changes in corolla shape and anther exsertion during the evolutionary history of the APSA clade compared to the more uniform floral bauplan of the rauvolfioids (Fishbein et al., 2018). These floral morphological changes can be viewed as pollinator‐driven adaptations, resulting in different pollination systems (Ollerton et al., 2019; see Discussion). Many of these floral trait changes are included in hypotheses by Bitencourt et al. (2021) on morphological synnovations (sequence of interacting trait innovations—sensu Donoghue and Sanderson, 2015), which have made it possible for Apocynaceae to diversify into more open, dry, and disturbed habitats. In addition to the floral and seed diversity, the wood anatomy of Apocynaceae was described in previous studies on rauvolfioids (Sidiyasa and Baas, 1998; Lens et al., 2008), apocynoids and Periplocoideae (Lens et al., 2009), and Secamonoideae and Asclepiadoideae (Singh, 1943; Patil and Rajput, 2008; Beckers et al., 2022). These authors identified several traits characteristic of (sub)tribes, such as the degree of vessel grouping, type and position of mineral inclusions, and the presence and distribution of axial parenchyma. Wood anatomical observations also supported hypotheses on the parallel evolution toward a climbing habit (Lahaye et al., 2005; Livshultz et al., 2007; Lens et al., 2009; Joubert et al., 2016; Fishbein et al., 2018; McDonnell et al., 2018), and showed that some anatomical traits are limited only to climbers, like the proportionally wide vessels and nonlignified zones in the wood cylinder that enable stem flexibility (Carlquist, 1985; Ewers et al., 1990; Gasson and Dobbins, 1991; Angyalossy et al., 2015). Although published studies on Apocynaceae wood anatomy provided valuable insights into tribal identification and climbing anatomy, an overview of wood evolution within the family remains missing. Considering the importance of wood anatomical traits to define higher‐level clades in the family (Lens et al., 2008, 2009), the occurrence of woody species in all major clades, and the presence of ancestrally woody species with various life‐forms as well as (phylogenetically) derived woody species that have evolved from a nonwoody ancestor (Beckers et al., 2022), the family Apocynaceae is perfectly suited for such a wood anatomical survey. More specifically, it would be interesting to investigate the evolutionary correlation of wood anatomical traits and morphological synnovations to explain the much higher diversification rates in later‐diverging Apocynaceae (Bitencourt et al., 2021), especially evolutionary changes in smaller growth‐forms with shorter generation times because these are regarded as adaptations to drier habitats (Smith and Donoghue, 2008). Therefore, we aimed to assess whether the gradual evolution of floral traits described above corresponds to gradual changes in wood anatomy, leading to an expanded set of synnovations essential for whole‐plant evolutionary processes in Apocynaceae.

The objectives of this study were (1) to summarize wood anatomical variation across Apocynaceae and reconstruct the evolution of wood anatomical traits, (2) to study the impact of evolutionary growth form transitions—among others, reduction in plant size of erect and scandent species and transitions from erect to climbing growth forms—on wood anatomical diversity, (3) to run correlation tests among and between anatomical and flower and seed traits to identify the presence of an expanded set of reproductive–vegetative synnovations.

## MATERIALS AND METHODS

### Wood sectioning, staining, and description

**Figure 2:**
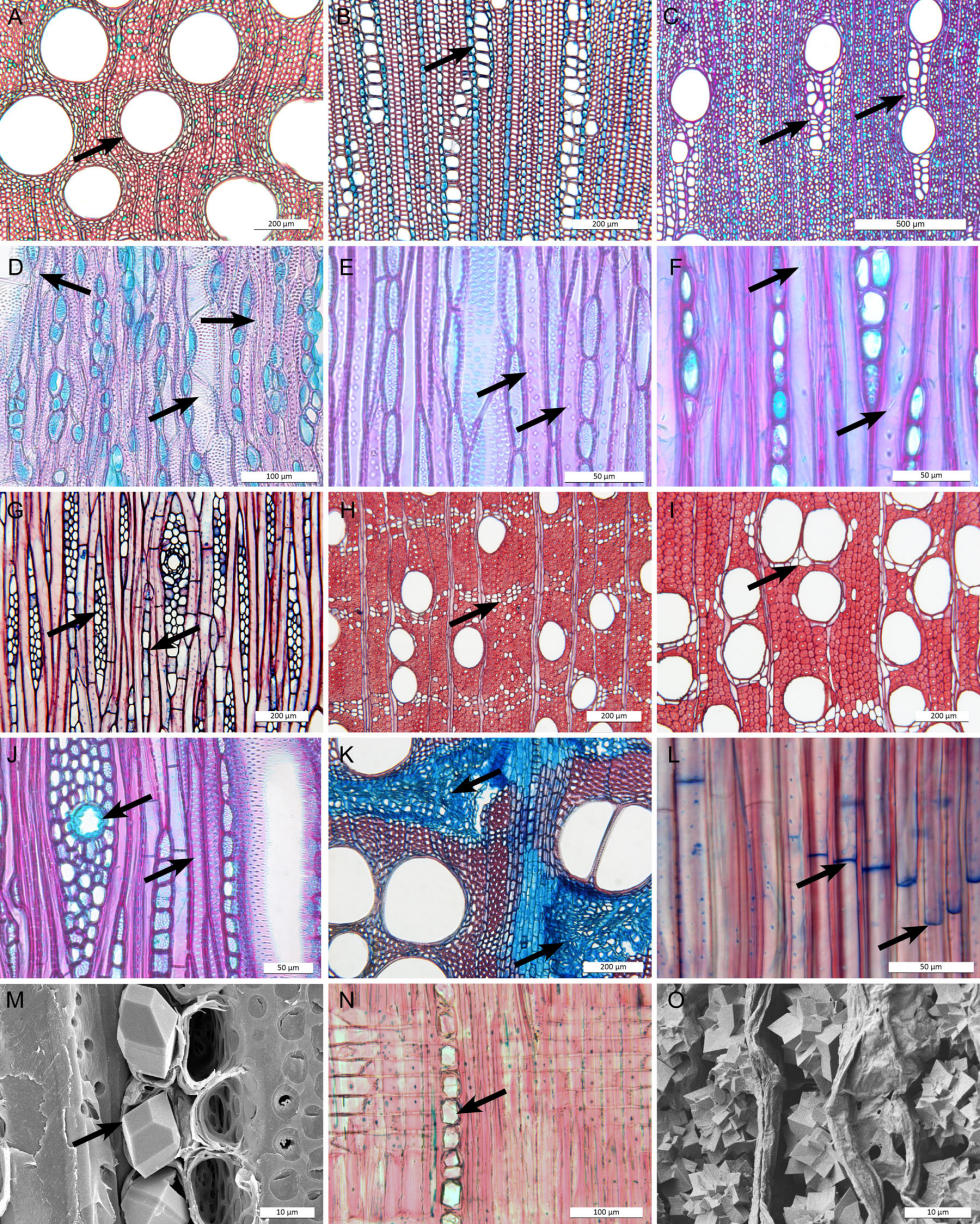
Overview of informative Apocynaceae wood anatomical characters based on LM sections (A–L, N) and SEM surfaces (M, O). (A) *Willughbeia coriacea—*cross section, mainly solitary vessels (arrow). (B) *Thevetia ahouai—*cross section, vessels mainly in radial multiples (arrow). (C) *Gongronemopsis tenacissima—*cross section, vessels in radially oriented clusters including many narrow (arrows) and few wide vessels (vessel dimorphism). (D) *Minaria acerosa—*tangential section, ground tissue composed of densely pitted imperforate tracheary cells (tracheids, arrow pointing right), which are difficult to distinguish from narrow vessels with simple perforations (tilted arrow pointing left) surrounding wider vessels (tilted arrow pointing right). (E) *Kanahia laniflora—*tangential section, ground tissue composed of fiber‐tracheids with distinctly bordered pits (arrows). (F) *Cerberiopsis candelabra—*tangential section, ground tissue composed of few minutely bordered libriform fibers (arrows point to pits). (G) *Dyera costulata—*tangential section, uni‐ (arrow pointing left), and multiseriate rays (arrow pointing right). (H) *Aspidosperma oblongum—*cross section, apotracheal axial parenchyma (arrow). (I) *Aspidosperma album—*cross section, paratracheal axial parenchyma (arrow). (J) *Secamonopsis madagascariensis—*tangential section, laticifer in multiseriate ray (arrow pointing left) and vasicentric tracheids (arrow pointing right). (K) *Leptadenia arborea—*cross section, cambial variant (arrows) leading to zones of nonlignified xylem (in blue) including fibers, parenchyma, rays, and traces of interxylary phloem cells. (L) *Mandevilla rugellosa—*radial section, septate libriform fibers (arrows pointing to septa). (M) *Pentopetia grevei—*tangential surface, prismatic crystals in ray cells (arrow). (N) *Aspidosperma megalocarpon—*radial section, prismatic crystals in chambered axial parenchyma cells (arrow), (O) *Funastrum clausum—*cross section, druses in nonlignified tissue.

A total of 275 wood anatomical species descriptions were collected or newly produced: 216 from the literature, 13 from InsideWood (InsideWood, 2004 onward; Wheeler, 2011), and 46 newly described. These descriptions cover all major clades of the family, except for Eustegieae and Fockeeae (both herbaceous), and most (96) woody genera—75% of the family is nonwoody—as recognized by Fishbein et al. (2018) and Endress et al. (2019). Wood samples were collected from the xylaria of Naturalis (Lw, Uw, WAGw), Tervuren (Tw), Kew (Kw), and Madison (MADw, SJRw), or generously donated by Apocynaceae experts (mentioned in Acknowledgments). Appendix S1 includes voucher information for the 46 newly described samples, and Appendix S2 gives an overview of references and taxonomic name changes for the 229 previously published wood descriptions (predominantly one sample per species, as is the standard in comparative wood anatomical studies). Newly described wood samples were sectioned, stained, and mounted according to the standard protocol of Lens et al. (2005) and described following the “IAWA list of microscopic features for hardwood identification” (IAWA Committee, 1989), with a few additions (see next paragraph). Previously published wood anatomical descriptions were also checked and adapted to correspond with the trait states used in this study. Below, short descriptions and illustrations are given for the most important Apocynaceae wood traits. We observed the sections with a Leica DM2500 light microscope (LM) equipped with a Leica DFC425 C digital camera. The LM and scanning electron microscopic (SEM) images for illustrative purposes (Figure 2) are from unpublished material of previous work.

### Definitions of informative wood anatomical traits

**Figure 3:**
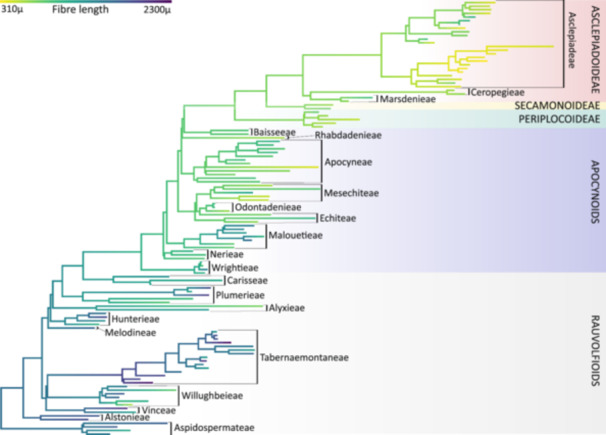
Maximum likelihood mapping of fiber length (in µm) on the phylogram of Fishbein et al. (2018).

To observe the three‐dimensional structure of wood, we studied three planes. Cross sections are most informative for quantitative vessel traits (e.g., vessel diameter and density), vessel grouping pattern, axial parenchyma distribution, and the presence of vascular cambiants sensu Cunha Neto (2023). Among these broadly circumscribed vascular variants, our study mainly deals with cambial variants as described by Carlquist (2001), which are defined as deviations from the single bidirectional vascular cambium, such as nonlignified zones in the wood cylinder that may have interxylary phloem, as observed in the wood of several climbing Apocynaceae. Other major vascular variant types in Apocynaceae are procambial variants (intraxylary phloem characterizing the entire family) and ectopic cambia (successive cambia only observed in *Odontadenia*; Lens et al., 2009). In tangential sections, ray width and height, intervessel pitting, and number of cells per axial parenchyma strand can be observed. Radial sections typically show cellular ray composition, vessel‐ray pitting, and vessel perforation plate morphology. Fiber‐pitting details and mineral inclusions are visible in tangential as well as radial‐orientated planes. Additionally, individual cell lengths (vessel elements, fibers) are measured in maceration slides, and vestures are typically observed with SEM. The definitions and illustrations (Figure 2) of the wood anatomical traits relevant for Apocynaceae wood evolution are as follows: *vessel diameter*—average tangential diameter (µm) of 25 vessels, which are dead hollow pipes consisting of separate cells (vessel elements) that axially connect to each other via perforation plates (Figure 2A–C); *vessel density*—average number of vessels (per mm^2^) measured for 10 regions in cross sections (Figure 2A–C), narrow vessels were excluded (standardized in IAWA list), except when mentioned otherwise; *vessel element length*—mean length (µm) of 25 cells (including the tails) measured in maceration slides, narrow vessels were excluded (standardized in IAWA list), except when mentioned otherwise; *vessel grouping*—we recognized three categories based on cross sections for the ancestral state reconstructions—(1) mainly solitary vessels (90% or more; Figures 2A), (2) radial multiples of 4 or more common (Figures 2B), (3) vessel clusters common (irregular groups of vessels, which can be radially oriented in case of vessel dimorphism; Figure 2C); *vessel dimorphism*—two distinct vessel diameter classes in diffuse‐porous wood (Figure 2C); *tracheids*—narrow and slender water‐conducting cells without perforation plates resembling narrow vessel elements in size, shape, and pitting, often vasicentric (surrounding vessels) (Figure 2D, J); *fiber‐tracheids*—narrow and slender (presumably nonconductive) cells with distinctly bordered pits which are smaller (3–6 µm) and fewer (mostly in radial, but sometimes also in tangential walls) compared to tracheids (Figure 2E); *libriform fibers*—narrow and slender (nonconductive) cells with simple to minutely bordered pits, of which the pit chambers are smaller (2–3 µm) and fewer (confined mainly to radial walls) compared to fiber‐tracheids (Figure 2F); *septate fibers*—fibers (mainly libriform) that are compartmentalized by thin septae (Figure 2L); *fiber length*—mean length (µm) of 25 fibers measured in maceration slides; *apotracheal axial parenchyma*: axial parenchyma (longitudinally oriented parenchyma cells containing water and metabolites) not associated with vessels as observed in cross sections (Figure 2H); *paratracheal axial parenchyma*—axial parenchyma associated with vessels (Figure 2I); *ray width*—based on tangential sections, we recognized two categories, (1) uniseriate rays that are one cell wide, and (2) multiseriate rays that are two or more cells wide (Figure 2G); *ray height*—mean height (µm) of 15 rays measured on tangential sections (Figure 2G); *mineral inclusions*—we recognized three categories, (1) prismatic—solitary rhombohedral or octahedral—crystals of calcium oxalate in ray cells (Figures 2M), (2) prismatic crystals in axial parenchyma cells (Figures 2N), (3) druses—star‐shaped compound crystals—in nonlignified xylem tissue (Figure 2O); *laticifers*—tubes in rays containing latex (Figure 2J); *vascular (cambial) variant*—any deviation from the normal bidirectional cambium activity that produces lignified secondary xylem inwards and secondary phloem outwards, in Apocynaceae represented by zones of nonlignified tissues in the wood cylinder that may contain traces of interxylary secondary phloem (Figure 2K). We excluded the presence of intraxylary phloem when scoring for vascular variations, because all Apocynaceae have this trait (though not visible in wood sections without the central stem part). For more background information on vascular variants (procambial variants, cambial variants, and ectopic cambia), see the works of Carlquist (2001); Pace et al. (2009, 2014), Angyalossy et al. (2015), Chery et al. (2020), and Cunha Neto (2023), and for Apocynaceae specifically, Lens et al. (2008, 2009) and Beckers et al. (2022).

### Phylogenetic comparative methods

We selected 17 potentially informative wood anatomical traits for ancestral state reconstructions and tests of correlated evolution among wood traits, and between wood traits and the floral and seed trait data set of Fishbein et al. (2018). Multistate wood traits are represented twice: once as polymorphic discrete characters that can be used for ancestral state reconstructions, and once where these characters are split into separate binary traits that could be used for the correlation tests (see Appendix S3 for the complete data set that includes the polymorphic and binary states of several traits). The time‐calibrated molecular chronogram and phylogram of Fishbein et al. (2018), with updated nomenclatural synonyms, were applied as phylogenetic framework. This framework of 1045 species (20–25% of the family) based on 21 chloroplast loci was pruned to match the species in the wood anatomical data set using the treedata function from the R package geigerv.2.0.10 (Pennel et al., 2014) in the R environment version 4.2.1 (R Core Team, 2022). Because single trees were available, state reconstructions of qualitative traits were done with stochastic mapping in R (see Joy et al., [2016] for a review on ancestral reconstruction methodologies and Revell and Harmon [2022] for phylogenetic comparative methods in R). Further functions mentioned below are from the R package phytools version 1.0‐3 (Revell, 2012). Ancestral state reconstruction models for discrete characters (“equal rates” and “all rates different”) were fitted with the fitMk function and compared with a log‐likelihood ratio test. Models for polymorphic discrete characters (“equal rates”, “symmetric backward & forward rates”, “all rates different”, and “transient”), were fitted with the fitpolyMk function and the model with the lowest Akaike information criterion (AIC) value was selected (see Appendix S4 for model selection of the different characters). Trait history calculations were estimated with the make.simmap function for 1000 simulations and a summary of the ancestral nodes (prior probabilities of each state at each node) was visualized using a script from Revell (2022; we refer readers to Holland et al. [2020] and Chapter 8 of Revell and Harmon [2022]) for discussion on different state reconstruction methods). Plotting of continuous characters on the phylogram was done with the contMap function, and testing for correlated evolution between binary traits was done with the fitPagel function (“equal rates” and “all rates different”). Colors were selected from the R package viridis v.0.6.2 (Garnier et al., 2021). The R script for the state reconstructions was adapted from Pace et al. (2022) and can be found on github.com/vickybeckers.

## RESULTS

### Anatomical summary and major evolutionary transitions in Apocynaceae wood

**Figure 4:**
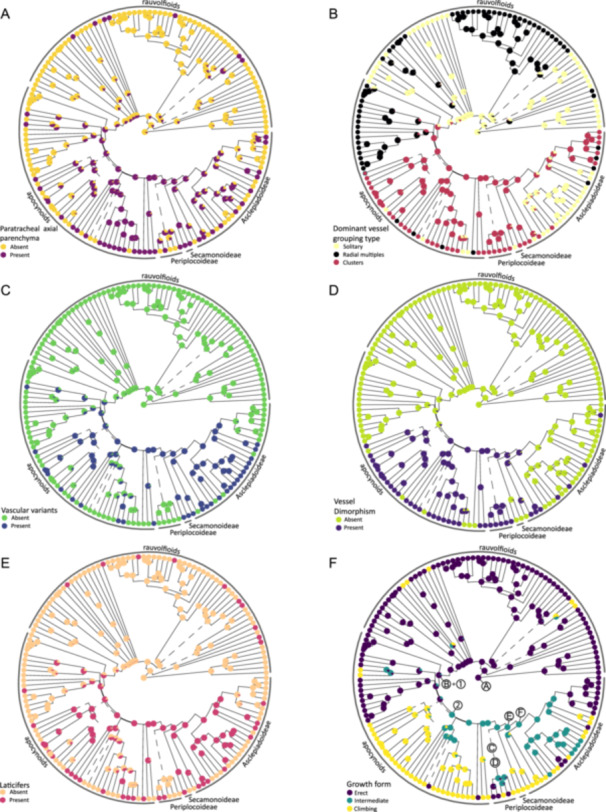
Ancestral state reconstructions with stochastic mapping using the chloroplast‐based chronogram of Fishbein et al. (2018) showing a suite of associated wood traits. Branches with less than 90% bootstrap support or indicates clades with conflicting arrangement are indicated with dashed lines (after support values in Fishbein et al., 2018). The chronogram only includes woody species for which anatomical traits were collected (all herbaceous species have been removed). Pie charts at the nodes represent prior probabilities for each state at each node. (A) Presence of paratracheal parenchyma (often in addition to apotracheal axial parenchyma). (B) Presence of dominant vessel grouping type. (C) Presence of vascular (cambia) variants. (D) Presence of vessel dimorphism. (E) Presence of laticifers in rays. (F) Woody growth‐form, important flower and seed traits A–F and 1–2 following Figure 1: A = stamens free of style head, B = gynostegium, C = scoop‐like translator with pollen in tetrads, D = scoop‐like translator with four pollinia, E = clip translator with four pollinia, F = clip translator with two pollinia, 1 = dry fruit with comose seeds, 2 = increase in climbing habit.

Apocynaceae woods are generally characterized by simple vessel perforation plates, alternate vestured intervessel pits, and vessel‐ray pits similar in shape and size to intervessel pits. Vessel arrangement varies from mainly solitary to long radial multiples and clusters, occasionally with vasicentric tracheids. Fibers vary from very thin‐ to very thick‐walled, and pits are either simple or distinctly bordered, occasionally septate. Axial parenchyma distribution varies from diffuse in aggregates, to banded, or positioned nearby vessels. Rays are uni‐ to multiseriate up to 12 cells wide. Laticifers and vascular (cambial) variants are regularly found, as are prismatic crystals in axial parenchyma and/or ray cells. Within subfamilies and grades, tribes can be identified based on a combination of the type and position of mineral inclusions, vessel grouping, axial parenchyma distribution, and ray width (Table 1). One example is the rauvolfioid clade Tabernaemontaneae. This tribe is unique within the family because of the presence of septate fibers (Figure 2L) combined with a scarcity of axial parenchyma. For Echiteae (apocynoids), Marsdenieae (Asclepiadoideae), and Periplocoideae, finding a set of defining wood anatomical traits proved unsuccessful, even after extending the previously published data sets with new species descriptions. In addition to the abovementioned set of traits, the reduction of fiber and vessel element length along the phylogenetic backbone of the family can help to assign an unknown wood sample to a specific lineage (Figure 3, see Appendix S5 for the ancestral state reconstructions not included in the main text). Furthermore, there is a general trend from predominantly apotracheal axial parenchyma in rauvolfioids and early‐branching apocynoids to more paratracheal axial parenchyma in the rest of the APSA lineages (Table 1, Figure 4A), with gradually fewer cells per strand (not illustrated). An increased presence of paratracheal axial parenchyma is associated with an increase of vessel clusters with vessel dimorphism, vascular (cambial) variants, and laticifers in the APSA clade, separating the wood of rauvolfioids and the early‐branching apocynoid tribes Malouetieae, Nerieae, and Wrightieae from the rest of the family (Figure 4A–E). Wood anatomical characters that are highly variable in the family are the distribution of apotracheal axial parenchyma (diffuse, diffuse in aggregates, or banded) and type of ground tissue (libriform fibers, fiber‐tracheids, or “true” tracheids; Appendix S5, see Appendix S6 for the correlation matrix).

**Table 1:** Summary of wood anatomical traits by tribe and subfamily/grade. Numbers in parentheses behind clade names are number of wood samples investigated (generally one sample per species with a few exceptions; see Appendices S1 and S2 for sampling specifics). Clades in grey are rauvolfioids, blue are apocynoids, green is Periplocoideae, yellow Secamonoideae, and red Asclepiadoideae. Tribes in bold include climbing species. +: present; ‐: absent; (): traits occasionally present but not found in all clade members.

	Vessels mainly solitary	Vessels mainly in radial multiples	Vessels mainly in clusters	Vessel dimorphism	(Vasicentric) tracheids common	Fiber‐tracheids	Libriform fibers	Septate fibers	Apotracheal axial parenchyma	Paratracheal axial parenchyma	Uniseriate rays	Multiseriate rays	Prismatic crystals in axial parenchyma	Prismatic crystals in ray cells	Vascular variants	Laticifers
Aspidospermateae (14)	+	‐	‐	‐	‐	+	(+)	‐	+	‐	‐	+	+	(+)	‐	‐
Alstonieae (5)	‐	+	‐	‐	‐	+	‐	‐	+	‐	‐	+	(+)	‐	‐	(+)
**Vinceae (13)**	+	(+)	‐	‐	‐	+	‐	‐	+	‐	+	+	+	+	?	‐
**Willughbeieae (26)**	+	+	(+)	(+)	+	+	‐	‐	+	+	+	+	(+)	‐	‐	+
Tabernaemontaneae (28)	+	+	‐	‐	‐	‐	+	+	‐	‐	+	+	‐	(+)	‐	‐
Diplorhynchus (2)		‐	‐	‐	‐	+	‐	‐	(+)	+	(+)	+	‐	+	‐	+
**Melodineae (4)**	+	(+)	‐	‐	+	+	‐	‐	+	+	+	+	(+)	+	‐	‐
Hunterieae (7)	+	‐	‐	‐	‐	+	+	‐	+	‐	+	+	‐	‐	‐	‐
**Amsonieae (2)**	+	‐	‐	‐	+	+	‐	‐	‐	‐	+	+	‐	‐	‐	+
**Alyxieae (10)**	+	‐	‐	‐	+	+	‐	‐	+	+	+	+	(+)	‐	‐	(+)
**Plumerieae (15)**	‐	+	(+)	‐	‐	(+)	+	(+)	+		+	+	‐	‐	‐	‐
Carisseae (5)	+	‐	‐	‐	‐	+	‐	‐	+	‐	+	+	+	‐	‐	‐
Wrightieae (8)	(+)	+	‐	‐	‐	+	+	‐	+	‐	+	+	‐	+	‐	‐
**Nerieae (10)**	‐	+	‐	‐	‐	(+)	+	‐	+	‐	‐	+	(+)	‐	(+)	(+)
**Malouetieae (16)**	‐	+	‐	‐	‐	(+)	+	‐	+	‐	+	+	+	‐	‐	‐
**Echiteae (6)**	‐	‐	+	+	+	(+)	+	‐	‐	+	+	+	(+)	(+)	(+)	+
**Odontadenieae (6)**	‐	‐	+	+	+	‐	+	+	‐	‐	‐	+	(+)	‐	‐	‐
**Mesechiteae (8)**	‐	‐	+	+	(+)	+	+	(+)	‐	+	+	+	+	+	(+)	+
**Apocyneae (20)**	(+)	‐	+	+	+	+	+	‐	+	+	‐	+	+	(+)	‐	+
**Rhabdadenieae (1)**	‐	+	‐	‐	‐	‐	+	‐	‐	+	+	‐	‐	‐	‐	+
**Baisseeae (6)**	‐	‐	+	+	+	+	+	‐	+	+	‐	+	+	(+)	‐	+
**Periplocoideae (8)**	+	‐	+	+	+	+	+	‐	+	+	+	+	+	(+)	(+)	‐
**Secamonoideae (9)**	+	‐	(+)	(+)	(+)	+	‐	‐	‐	‐	‐	+	(+)	(+)	‐	(+)
**Marsdenieae (21)**	+	‐	+	+	‐	+	+	‐	‐	+	+	+	‐	‐	(+)	(+)
**Ceropegieae (6)**	+	‐	(+)	(+)	‐	+	‐	‐	‐	+	+	+	‐	+	+	+
**Asclepiadeae (38)**	+	+	+	(+)	(+)	+	+	‐	‐	+	+	‐	‐	‐	(+)	(+)

### Impact of evolutionary growth‐form transitions

Some wood anatomical variation can be explained by a difference in growth‐form, such as the presence of wider vessels in lianas compared to erect species of similar height (Table 2). Intermediate growth‐forms—e.g., twining shrubs—are mostly present in later‐branching representatives of the APSA clade, such as *Periploca*, *Secamone*, and Metastelmatineae. This plasticity in growth‐form can also be seen within a single plant when a plant starts with an erect growth habit but climbs as soon as it finds a host. Developmental changes from erect to climbing are well known in Asclepiadoideae, such as *Vincetoxicum sylvaticum* and *Matelea pedalis* (Beckers et al., 2022). In the erect, initial growth stage, there is a continuous ring of metaxylem consisting of very long and narrow vessel elements and parenchyma in radial rows (Figure 3 of Beckers et al., 2022). Secondary growth follows with wider vessels within a largely nonlignified stem during the later climbing stage. Despite this plasticity, there are two distinct basic bauplans in Apocynaceae climbers: (1) rauvolfioids and early‐branching apocynoids have solitary vessels with densely pitted imperforate ground tissue cells and an absence of nonlignified zones in the wood cylinder, and (2) later‐branching APSA members have vessel clusters with vessel dimorphism, often vascular (cambial) variants, and a variable imperforate ground tissue (Table 2). This structural change in wood anatomy seems to have evolved separately from the estimated four independent evolutionary transitions between a climbing and erect habit (Figures 1, 4F). Another plastic growth‐form trait is plant size, which dramatically decreases along the backbone of the phylogeny and matches the shortening of the fibers and vessel elements (Figure 3; Appendix S5). However, this correlation with plant size is not as clear as the tight correlation between vessel element and fiber length, which is not surprising given that the last two share the same developmental origin from a so‐called fusiform cambium cell (Appendix S6).

**Table 2:** Comparison between wood anatomical traits of climbing and erect species within major Apocynaceae groups. Tribes in grey are rauvolfioids, red represents Asclepiadoideae, and green is Periplocoideae. Numbers in parentheses behind clade names are the number of wood samples investigated (generally one sample per species with a few exceptions; see Appendices S1 and S2 for sampling specifics). +: present; –: absent; (): traits occasionally present but not found in all clade members. * (probably) includes intermediate growth forms, ** includes erect *Diplorhynchus*. Vessel dimensions exclude narrow vessels in case of vessel dimorphism.

		Range of average vessel diameter (µm)	Range of average vessel element length (µm)	Species with mainly solitary vessels	Species with mainly vessels in radial multiples	Species with mainly vessels in clusters	Vessel dimorphism	Range of average fiber length (µm)	Laticifers	Vascular variants
**Alyxieae**	**Erect (2)**	60	480‐1370	+	+	‐	‐	1240‐2980	‐	‐
	**Climbing (10)**	80‐220	430‐940	+	‐	‐	‐	730‐1740	(+)	‐
Melodineae**	**Erect (3)**	40‐70	550‐710	+	‐	‐	‐	1420‐1630	+	‐
	**Climbing (3)**	70‐130	470‐720	+	‐	‐	‐	1050‐1150	‐	‐
**Willughbeeae**	**Erect (8)**	50‐120	430‐1020	‐	+	‐	‐	920‐1700	‐	‐
	**Climbing (17)**	60‐230	410‐630	+	‐	(+)	(+)	580‐1690	+	‐
**Periplocoideae**	Erect (4*)	40‐50	225‐320	+	‐	‐	‐	540‐600	‐	‐
	**Climbing (4)**	100‐240	250‐450	+	‐	+	(+)	500‐850	‐	+
**Asclepiadeae**	Erect (16*)	30‐90	160‐570	+	+	+	(+)	340‐1110	(+)	‐
	**Climbing (16)**	60‐190	250‐500	+	‐	+	+	420‐740	(+)	+

Attempts to categorize the complex variation in the ground tissue (fiber‐tracheids, libriform fibers and “true” tracheids) did not result in any correlations with habit or other wood traits, and they were widely distributed across the phylogeny (Table 1; Appendices S5). Pit size and density on the tangential and/or radial walls of imperforate tracheary cells, which were used for defining the traits, showed considerable variation, making it impossible to assign clear boundaries for discrete character states. An extreme case of imperforate tracheary element variation was observed in one of the lightest, styrofoam‐like woods: *Alstonia spatulata* (see excellent photos and microscope images by Baas et al., 2019). In this swamp forest tree species, the trunk base and root collar have “tracheids” that lack any intrusive tip growth and have numerous vestured pits in which the pit borders have been fully reduced, whereas the wood higher in the trunk and branches is composed of “normal” fiber‐tracheids with fully bordered pit pairs and intrusive tip growth (Sidiyasa and Baas, 1998; Baas et al., 2019).

### Evolution within and between wood anatomical and floral and seed traits

Almost all of the wood anatomical traits investigated were found to be phylogenetically informative (Appendix S4). The rauvolfioids and early‐branching apocynoids are separated from the rest of the APSA clade by a scarcity or absence of vessel clusters and vessel dimorphism, vascular (cambial) variants, and laticifers in rays. Many of the informative wood traits also changed together with a selection of morphological traits (Appendix S6), without invoking any causality. For example, the more dominant presence of (homoplasious) fleshy fruits in the rauvolfioids co‐occurs with rare or absent apotracheal axial parenchyma, which is opposite to the dry fruits and paratracheal axial parenchyma dominant in the APSA clade. The presence of a gynostegium (anthers attached to the style head; Figure 1)—arguably the most important morphological difference between the rauvolfioids and APSA clade—did not correlate much with wood traits, which is not surprising since the cascade of wood anatomical trait shifts builds up in the later branching groups of the APSA clade. The suggested correlation of septate wood fibers, a salverform corolla and fleshy fruits at similar nodes in the phylogeny may be misleading since these traits typically represent Tabernaemontaneae species, which were overrepresented in the rauvolfioid data set. Several wood traits associated with APSA members (e.g., vessels in clusters, vascular (cambial) variants, and laticifers) correlated with flower, seed, and fruit traits in APSA evolution (e.g., dry fruits, pollinia, and a gynostegium—equal rates model; Appendix S6). These APSA species outnumbered species in the rauvolfioid and early‐branching apocynoids, which may have skewed the correlation tests as well. Least correlated wood anatomical traits were (binary) traits that are informative within a set of synapomorphies that define tribes, such as the presence and position of prismatic crystals.

## DISCUSSION

### Wood anatomical diversity and evolution across Apocynaceae

Several wood anatomical characters are informative at different taxonomic levels. Across all Gentianales families, vestured intervessel pits are always observed (e.g., Metcalfe and Chalk, 1950; Meylan and Butterfield, 1974; Jansen and Smets, 1998; Jansen et al., 2001) as is intraxylary phloem (except for Rubiaceae; Metcalfe and Chalk, 1950; Carlquist, 1984; Lens et al., 2008, 2009; Dalvi et al., 2014; Beckers et al., 2022). Within Apocynaceae, major groups can be recognized by a number of wood anatomical traits (Table 1; Metcalfe and Chalk, 1950; Lens et al., 2008, 2009; Beckers et al., 2022). For example, the rauvolfioids are typically identified by extensive radial vessel multiples in erect species (Figure 2B) and solitary vessels in climbers (Figure 2A). Within this grade, Tabernaemontaneae is unique because of the presence of septate fibers (Figure 2L) and scarce axial parenchyma, two traits that do not co‐occur outside this tribe (Lens et al., 2008). Wood anatomy is also informative to distinguish the rauvolfioids and early‐branching apocynoid tribes Malouetieae, Nerieae, and Wrightieae from the rest of the APSA clade (core apocynoids, Periplocoideae, Secamonoideae, and Asclepiadoideae). In this remaining APSA clade, there is an evolutionary transition toward more paratracheal axial parenchyma, anomalies during vascular development (vascular variants, which include cambial variants; Figure 2K; Lens et al., 2009; Beckers et al., 2022; Cunha Neto, 2023), gradually shorter vessel elements and fibres (Figure 3; Appendix S5), and more laticifers in rays (Figure 2J). Even though ancestral reconstructions suggest that laticifers originated earlier in the evolution of Apocynaceae (Figure 4E), the rare occurrence of laticifers in wood makes it hard to convincingly score this trait for a given Apocynaceae species. Additionally, vessel clustering becomes more common, often in combination with vessel dimorphism—the co‐occurrence of few wide and many narrow vessels (Figure 4B, D; Table 1; Lens et al., 2009; Beckers et al., 2022).

Major (sub)tribes within the APSA clade also can be identified by wood anatomical characters. For example, two Asclepiadoideae subtribes have a unique set of characters: Leptadeniinae have tall multiseriate rays up to 12 cells wide and islands of nonlignified tissue in the wood cylinder, and Metastelmatinae show furrowed nonlignified wood tissue and very thick‐walled xylem fibers surrounding mainly solitary vessels (Beckers et al., 2022). Vessel clustering in Marsdenieae and Asclepiadinae (Asclepiadeae; Figure 1) is shared with apocynoids and Periplocoideae, but Secamonoideae and most Ceropegieae and Asclepiadeae subtribes have predominantly solitary vessels (Figure 4B; Appendix S5; Beckers et al., 2022). A few of the trait reconstructions in the rauvolfioids and early‐branching apocynoids are supported by reliable fossil descriptions (*Aspidospermoxylon uniseriatum* [Eocene], *Parahancornioxylon piptadiensis* [Pliocene], and *Tabernaemontana moralesii* [late Middle Eocene]; InsideWood, 2004 onward; Woodcock et al., 2017; Elisabeth Wheeler, North Carolina State University, personal communication). Unfortunately, the identification of *Apocynoxylon sylvestris* (Eocene), the only fossil presumably belonging to the more recently diverged groups of the APSA clade, is questionable because of its septate fibers (Gazeau and Koeniguer, 1975), which are absent in those species that we observed or are described in the literature (Table 1).

### Impact of plant size reduction, and transitions to a climbing and succulent habit

General plant size reduction is one major evolutionary trend along the phylogenetic backbone of the family (Bitencourt et al., 2021). This size reduction corresponds to a gradual shortening of fibers and vessel elements as a result of shorter fusiform cells in the vascular cambium (Figure 3; Appendices S5) and is also associated with reduced vessel width and habitat shifts (e.g., Baas et al., 1983; Olson et al., 2014; Echeverría et al., 2023). It is remarkable to observe this trend of decreasing fiber and vessel element length, despite three sources of uncertainty in our data set: (1) Plant size may be unrealistically represented in our study because we had to use maximum plant size values found in the literature since plant height was often not mentioned for the individuals sampled, especially for climbing species. (2) Many of our xylarium wood samples do not include information on the exact position of the wood sampled (trunk vs mature or younger branch). (3) Mostly only one (mature) wood sample per species is included in our study, meaning that some of the rare traits (e.g., laticifers in wood) may be scored as absent and intraspecific variation of quantitative traits for species and population is not covered (e.g., Hajek et al., 2016; Hacke et al., 2017; García‐Cervigón et al., 2020). Another impact of plant size reduction—and hence reduction of secondary growth—is the shorter life span leading to higher molecular rates of evolution (Smith and Donoghue, 2008), which facilitated invasion into new (often drier) environments (e.g., Givnish, 2010) that could have triggered speciation (Bitencourt et al., 2021; Figure 1). Interestingly, Beckers et al. (2022) estimated over 30 transitions from herbaceousness back to (phylogenetically derived) woodiness in Asclepiadoideae (Figure 1; Fishbein et al., 2018; Endress et al., 2019; Beckers et al., 2022), potentially as a response to (periodic) droughts (Lens et al., 2013; Zizka et al., 2022). Despite these independent transitions, the wood anatomy of these small woody lineages matches with the ancestrally woody APSA relatives, and nicely follows the general length reduction of quantitative traits across the family (e.g., Figure 3). The impact on the wood anatomy as a result of the independent woody growth form transitions from erect to climbing habit is more pronounced: climbers of the rauvolfioids and early‐branching apocynoids have mostly wide solitary vessels embedded in a ground tissue of densely pitted imperforate tracheary elements, whereas the rest of the climbing APSA clade show wide vessels associated with many narrow vessels in dense clusters in combination with nonlignified wood zones (Lens et al., 2009; Figures 2, 4). Although the anatomical bauplan of Apocynaceae suggests two independent transitions from erect toward climbing, assessing the true number of shifts is much more complicated due to the many intermediate life forms (e.g., Lens et al., 2009).

The nonlignified wood zones described in the results are essential for the climbing growth form to ensure stem flexibility and are often accompanied by an increased presence of axial parenchyma or cambial variants (Angyalossy et al., 2015). In Asclepiadoideae, the shape of the nonlignified zones is even specific to (sub)tribes (Beckers et al., 2022). However, we can also speculate that having a wood cylinder with increased parenchymatic zones can help the plant survive recurrent episodes of drought stress by storing more water (Carlquist and DeVore, 1998; Carlquist, 2001). For further understanding the impact of drought on the wood anatomy of Apocynaceae, additional functional studies can be done to measure capacitance in stems for climbers with nonlignified zones in the wood cylinder and for species that developed a succulent stem with an abundance of poorly lignified cells in the wood cylinder (e.g., many Ceropegieae; however, the wood of only very few succulent species has been anatomically described—see Liede and Kunze [2002], Mauseth [2004]). Also unclear is how the huge variation in imperforate tracheary element types (water‐conducting tracheids versus supposedly nonconducting fiber‐tracheids and libriform fibers) and its many intermediate forms in the APSA climbers correspond to the climatic niche of the species and hence the function of these cells in the root‐to‐shoot water transport pathway. Our standardized categorization does not show any phylogenetic signal (see Appendix S4 for results on phylogenetic signal testing) or correlation with other traits (see Appendix S6 for correlation matrix of Pearson's test for correlated evolution). Hydraulic experiments that stain only the functional water‐conducting cells at different drought stress levels will shed more light on how to fine‐tune the descriptive continuous categorization of the imperforate tracheary elements into more clear‐cut functional trait classes (e.g., Braun, 1970; Sano et al., 2005; Jupa et al., 2015).

### Changes in wood anatomy and flower morphology are linked in a surge of evolutionary innovations

In addition to the observed wood anatomical changes over time, a series of flower and seed trait changes occurred during the evolutionary history of Apocynaceae. In rauvolfioids, the stamens with pollen in monads are free of the style head, whereas they are attached in apocynoids (usually by agglutination) and in Periplocoideae, Secamonoideae, and Asclepiadoideae (by cellular fusion). This cellular fusion led to a rigidly fixed and canalized pentamerous revolver flower system that forces pollinators to enter through one of five narrow chambers, where they pass an acellular translator (scoop with sticky pad in Periplocoideae, clip in Secamonoideae and Asclepiadoideae; Figure 1) secreted by the style head. The translator is precisely alined with either free pollen in tetrads (Periplocoideae), or highly evolved pollen units (pollinia—four in Periplocoideae and Secamonoideae, two in Asclepiadoideae; Figure 1), which are attached to the pollinator when exiting the flower. The evolutionary explosion of floral morphological variation co‐occurred with a shift in pollinators within the APSA clade. Apocynoid flowers are pollinated mainly by bees and hawkmoths, similar to the rauvolfioids, whereas the rest of the APSA clade is mainly pollinated by flies and wasps (Ollerton et al., 2019). This transition toward insects that have shorter mouth parts and are less dependent on flowers to fulfill their life cycle probably tapped into previously unoccupied niches (Ollerton et al., 2003) and together with the evolution of pollinia and translators can be seen as an additional step in the synnovation that enabled the burst of species diversification that characterize the later‐branching Apocynaceae (Livshultz et al., 2011, 2018; Bitencourt et al., 2021).

There is a shift in wood anatomy within the apocynoids (see first paragraph, Discussion) that associates well with flower and seed adaptations (Appendix S6) and happens at similar topological positions in the phylogeny (Figure 4). This suite of extensive clusters of vessels with two distinct width classes, together with vascular (cambial) variants that enable stem flexibility and water storage, a variable imperforate ground tissue, and reduced wood cylinders with associated shorter life span (Table 2) probably increased survival of these lineages after the early Eocene climatic optimum, when temperatures dropped and seasonality increased (Bitencourt et al., 2021: see paragraph on drought adaptations). Consequently, these changes in wood anatomy offer additional potential drivers of diversification on top of the innovations leading to a climbing habit in the apocynoids (Bitencourt et al., 2021; Figure 1). In the rauvolfioids, the Tabernaemontaneae stand out. It is the only rauvolfioid tribe with a relative high speciation rate in comparison to other rauvolfioids, probably due to a shift from fleshy berries to dehiscent fruits with arillate seeds (Bitencourt et al., 2021). Coinciding with these morphological changes is a unique set of wood traits that include septate fibers that replaced the scarce apotracheal parenchyma (Table 1; Lens et al., 2008).

Modeling trait evolution and diversification rates in Apocynaceae is rather challenging for several reasons. A first reason is that chloroplast‐based molecular phylogenies provide low support for several internal nodes leading to tribes (Baisseeae, Echiteae, Mesechiteae, Odontadenieae, Apocyneae, Rhabdadenieae) and even subfamilies (Periplocoideae). Similar low support values for some key clades are also retrieved in nuclear‐based phylogenomic approaches that add significantly more molecular markers compared to the traditional Sanger sequencing (Antonelli et al., 2021). For example, the placement of Periplocoideae is unresolved in both chloroplast and nuclear analyses, which is often explained as an artefact due to missing data (Simmons, 2014; Fishbein et al., 2018) and incomplete lineage sorting (e.g., McDonnell et al. [2018] for Gonolobinae), although known problems such as hybridization (e.g., Morales‐Briones et al., 2018) and chloroplast capture (e.g., ter Steege et al., 2023) remain understudied in the family. Additional, more specific reasons why wood anatomical traits are difficult to link to non‐anatomical synnovations are incomplete sampling (the overlapping 146 species that represent only 10% of woody Apocynaceae; Figure 1), and the impact of growth‐form on wood anatomical traits (Figure 3, Table 2). Likewise, correlated evolution analyses are limited because they are only possible for binary states, thereby ignoring informative multistate trait variation. However, despite these restrictions, we found strong evidence for a cascade of morphological (flower, seed, growth form) and wood anatomical trait changes at neighboring nodes in the family's phylogeny that provide a more complete picture of how this synnovation contributed to the current species diversity in Apocynaceae, without invoking causation or clues about directional evolution. Indeed, it is unlikely that there is a causal explanation for the co‐occurring vegetative and reproductive trait changes over evolutionary time, because the vegetative traits are more likely driven by changes in environmental conditions (Carlquist, 2001), while the reproductive traits are better explained by changes in pollinator type (Ollerton et al., 2019). However, different plant parts are linked, and water availability is essential for flowers (McMann et al., 2022), especially since they are more vulnerable to xylem cavitation during drought than leaves (Zang and Brodribb, 2017).

## CONCLUSIONS

Apocynaceae exhibit a wide variation in wood anatomical traits. Part of this variation is due to the impact of growth‐form transitions, ranging from tall to short, erect to climbing, or woody to herbaceous. Another part of the wood anatomical variation is driven by evolution, allowing us to define several higher‐level clades ([sub]tribes, subfamilies) within the family. Examples of these phylogenetically informative wood traits are the presence and location of prismatic crystals, vessel grouping, variation in ray width, and anomalies in the wood cylinder that are the result of vascular variants, including the most common cambial variants in Apocynaceae. These vascular variants are more common in later‐branching woody Apocynaceae than in the rauvolfioids and early‐branching apocynoid tribes Malouetieae, Nerieae, and Wrightieae, and converge at more or less the same topological position in the phylogenetic tree with transitions toward other wood anatomical traits, such as more pronounced vessel multiples that include few large and many narrow vessels, more paratracheal axial parenchyma, and more laticifers in rays. Without any causal link invoked, this coordinated suite of wood anatomical traits in the later‐branching Apocynaceae evolved together with a burst of highly specialized flower traits and a reduced life span or shortened life cycle and gave rise to a spectacular synnovation event that led to the most species‐diverse clades in the family.

## AUTHOR CONTRIBUTIONS

F.L. and V.B. designed the research. F.L. and V.B. collected the samples. F.L. and V.B. conducted the laboratory work and collected the data. V.B. analyzed the data with supervision of M.E., P.B., E.S., and F.L. All authors interpreted the results. V.B. wrote the manuscript with supervision and participation by M.E., P.B., E.S., and F.L.

## Supporting information


**Appendix S1.** Voucher information of newly described wood species.

**Appendix S2.** Species name updates based on recent publications and references to previously described wood species.

**Appendix S3.** Data table of 17 wood traits and adaptations to the flower, seed, and fruit trait data set of Fishbein et al. ( 2018) that was used for ancestral state reconstructions and tests for correlated evolution.

**Appendix S4.** Results of the phylogenetic informativeness tests, and model selection for ancestral state reconstructions.

**Appendix S5.** Visualization of ancestral state reconstructions with species names as tip labels and reconstructions not included in the main text.

**Appendix S6.** Result matrix of tests for correlated evolution within and between anatomical and flower, seed, and fruit traits.

## Data Availability

All new and previously sectioned microscopic slides available are incorporated into the collections of Naturalis Biodiversity Center. Voucher information with searchable accession number can be found in Appendix S1. The full data table (Appendix S3) used for analyses can also be found on the Github repository of V.B.: https://github.com/vickybeckers.
