# Supplementary material for: Apocynaceae wood evolution matches key morphological innovations
Source: Am J Bot. 2024 Nov 22;111(11):e16436. doi: 10.1002/ajb2.16436 (PMC11584039; doi:10.1002/ajb2.16436)
Supplement: Supplementary file 4 — Appendix S4. Results of the phylogenetic informativeness tests, and model selection for ancestral state reconstructions. [file AJB2-111-e16436-s006.docx]

**Beckers et al.—American Journal of Botany 2024—Appendix S4.** Results of the phylogenetic signal test, correlation test of continuous traits, and model selection for the ancestral state reconstructions. AP = Axial parenchyma, APOPARA = Apotracheal and/or paratracheal axial parenchyma, BAN = Banded apotracheal axial parenchyma, CL = Mainly vessels in clusters, Clim = Climbing yes or no, DEN = Average vessel density (/mm^2), DIA = Diffuse in aggregates apotracheal axial parenchyma, DIAM = Average vessel diameter (µm), DIF = Diffuse apotracheal axial parenchyma, FCT = Fibre cell wall thickness, FL = Average fibre length (µm), FT = Fibre-tracheids, GF = Growth form, GTT = Ground tissue type, LA = Laticifers, LF = Libriform fibres, MI = Mineral inclusions, MPH = Maximum plant height (m), MT = Vessel multiple type (absent = mainly solitary), MUL = Vessel multiples present or absent, PC = Prismatic crystals, PCA = Prismatic crystals in axial parenchyma, PCP = Prismatic crystal position, PCR = Prismatic crystals in rays, RH = Average ray height (µm), RM = Mainly vessels in radial multiples, RW = Ray width (Uni = uniseriate - one cell wide, Multi = multiseriate - two or more cells wide), SCA = Scanty paratracheal parenchyma, SEP = Septate fibres, SOL = Mainly solitary vessels, T = Tracheids, VAS = Vasicentric paratracheal parenchyma, VD = Vessel dimorphism, VEL = Average vessel element length (µm), VV = Vascular variants.

Phylogenetic signal

fitContinuous function Geiger package: “lambda is one of the Pagel (1999) models that fits the extent to which the phylogeny predicts covariance among trait values for species. The model effectively transforms the tree: values of lambda near 0 cause the phylogeny to become more star-like, and a lambda value of 1 recovers the Brownian motion model.” Lambda = 1 is same as BM model, lambda = 0 is no phylogenetic signal. 0-= no signal, 0-0.3 = weak (not observed), 0.3-0.8 = moderate, 0.3- >0.8 = strong. Categories based on Münkemuller, T., S. Lavergne, B. Bzeznik, S. Dray, T. Jombart, K. Schiffers, and W. Thuiller. How to measure and test phylogenetic signal. *Methods in Ecology and Evolution* 3: 743-756. Doi: 10.1111/j.2041-210X.2012.00196.x. For discrete characters a comparison needs to be made between ER, ARD, or SYM. Model with lowest AIC value was chosen. ARD and SYM give sometimes same AIC value, ARD was then chosen. Traits in bold are used for the pairwise correlation test. *These values only make sense when you compare between models in the fitContinuous function (alternatives are BM, OU, EB, rate_trend, kappa, delta, mean_trend, white).

| **Trait** | **Nr. obs.** | **Model** | **Pagel’s lambda** | **Log-likelihood*** | **AIC*** | **AICc*** |
| --- | --- | --- | --- | --- | --- | --- |
| FL | 119 | Lambda | 0.651058 | -862.758211 | 1731.516422 | 1731.725118 |
| VEL | 127 | Lambda | 0.608759 | -835.270695 | 1676.541391 | 1676.736513 |
| **DIAM** | **134** | **Lambda** | **0.982006** | **-671.760671** | **1349.521342** | **1349.705958** |
| DEN | 134 | Lambda | 0.724487 | -649.896503 | 1305.793005 | 1305.977621 |
| RH | 134 | Lambda | 0.334533 | -959.160607 | 1924.321214 | 1924.505829 |
| MPH | 138 | Lambda | 0.719034 | -545.509094 | 1097.018189 | 1097.197293 |
| CL | 144 | ER | 1.000000 | -49.742262 | 103.484525 | 103.569631 |
| **GF** | **147** | **ARD** | **1.000000** | **-61.072923** | **136.145846** | **136.951601** |
| SOL | 144 | ARD | 0.663447 | -85.377210 | 176.754420 | 176.925848 |
| **MT** | **144** | **ARD** | **0.882964** | **-93.212701** | **200.425402** | **201.248932** |
| MUL | 144 | ER | 0.941800 | -62.073008 | 128.146016 | 128.231122 |
| **VD** | **144** | **ARD** | **0.823995** | **-49.044864** | **104.089729** | **104.261157** |
| T | 146 | ER | 1.000000 | -42.296749 | 88.593498 | 88.677415 |
| FT | 144 | ARD | 0.693602 | -81.020884 | 168.041768 | 168.213196 |
| LF | 144 | ARD | 0.701663 | -77.685754 | 161.371508 | 161.542936 |
| **GTT** | **144** | **ER** | **0.839641** | **-186.058049** | **376.116099** | **376.201205** |
| SEP | 147 | ER | 0.546718 | -37.208859 | 78.417717 | 78.501051 |
| VCT | 147 | SYM | 0.517166 | -126.399973 | 266.799946 | 267.605701 |
| AP | 147 | ER | 0.560985 | -75.209607 | 154.419214 | 154.502547 |
| APOPARA | 147 | ARD | 0.688402 | -150.583055 | 327.166111 | 329.902953 |
| DIF | 147 | ARD | 0.471375 | -37.005028 | 80.010055 | 80.177887 |
| **DIA** | **147** | **ARD** | **0.919429** | **-77.119952** | **160.239904** | **160.407736** |
| BAN | 147 | ER | 0.678563 | -83.712447 | 171.424894 | 171.508228 |
| SCA | 147 | ARD | 0.578128 | -79.875391 | 165.750782 | 165.918614 |
| VAS | 147 | ER | 0.000000 | -18.361631 | 40.723261 | 40.806595 |
| RW | 147 | ARD | 0.717603 | -123.481586 | 260.963172 | 261.768927 |
| PC | 147 | ER | 0.937911 | -97.227474 | 198.454949 | 198.538282 |
| **PCP** | **147** | **SYM** | **0.951220** | **-168.403566** | **362.807131** | **365.543973** |
| MI | 147 | SYM | 0.534124 | -132.394705 | 290.789410 | 293.526252 |
| **VV** | **147** | **SYM** | **1.000000** | **-44.542626** | **95.085251** | **95.253083** |
| **LA** | **147** | **ARD** | **0.989704** | **-77.679938** | **161.359875** | **161.527707** |

Correlation tests of continuous traits

Pairwise correlation tests done with pairs.panels function of psych package in r. Values above the diagonal histograms are the Pearson correlation values. *, **, and *** are different levels of significance. DIAM = vessel diameter (µm), DEN = vessel density (/mm^2^), VEL = vessel element length (µm), FL = fibre length (µm), RH = ray height (µm), MPH = maximum plant height (µm).


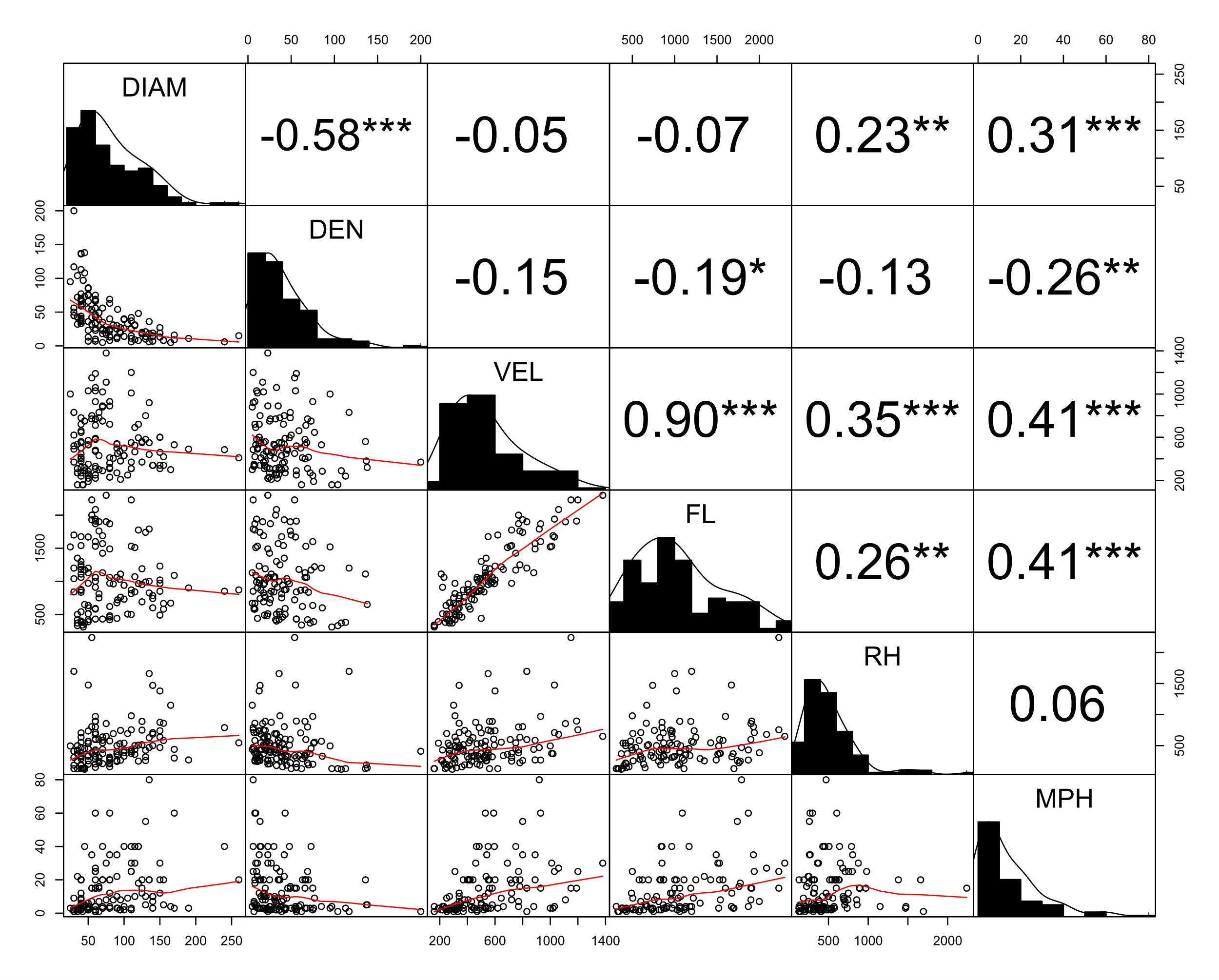


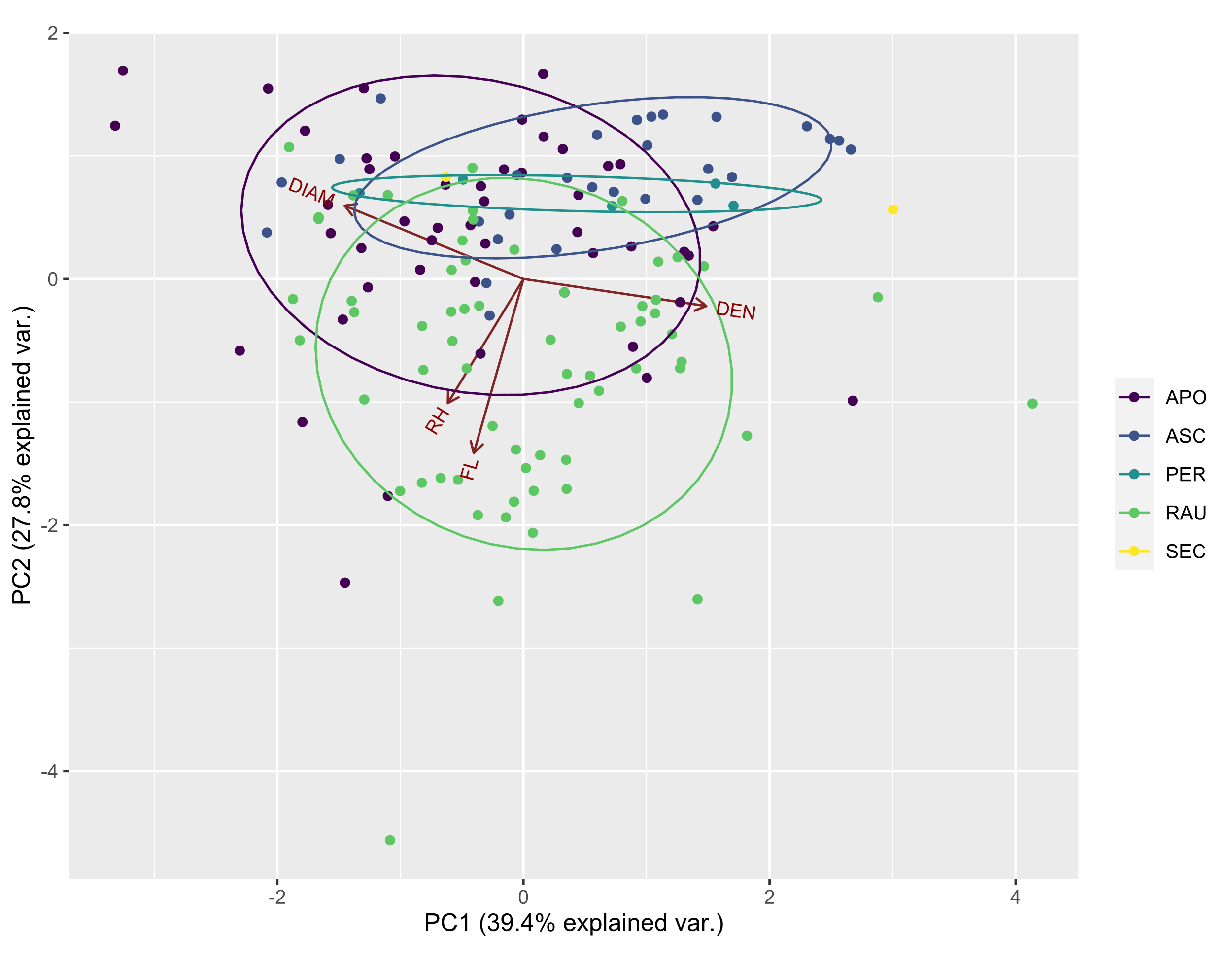


PCA of continuous traits.

Model selection of ancestral state reconstruction: discrete traits

Log-likelihood ratio comparison between ‘equal rates’ (ER) and ‘all rates different’ (ARD) models. When p-value is below 0.05 ARD model is chosen for stochastic mapping.

| **Trait** | **States** | **P-value** | **Model chosen** |
| --- | --- | --- | --- |
| Solitary vessels | Present, Absent | 0.04 | ARD |
| Vessel multiples | Present, Absent | 0.57 | ER |
| Vessel dimorphism | Present, Absent | 0.00 | ARD |
| Tracheids | Present, Absent | 0.40 | ER |
| Fibre-tracheids | Present, Absent | 0.16 | ER |
| Libriform fibres | Present, Absent | 0.04 | ARD |
| Septate fibres | Present, Absent | 0.19 | ER |
| Fibre cell wall thickness | Thin, Thin to Thick, Thick | 0.00 | ARD |
| Axial Parenchyma | Present, Absent | 0.00 | ARD |
| Diffuse apotracheal | Present, Absent | 0.00 | ARD |
| Diffuse in aggregates | Present, Absent | 0.54 | ER |
| Banded apotracheal | Present, Absent | 0.00 | ARD |
| Scanty paratracheal | Present, Absent | 0.01 | ARD |
| Vasicentric paratracheal | Present, Absent | 0.00 | ARD |
| Prismatic crystals | Present, Absent | 0.36 | ER |
| Mineral inclusions | Absent, Prismatic crystals, Druses, Various | 0.00 | ARD |
| Vascular variances | Present, Absent | 0.00 | ARD |
| Laticifers | Present, Absent | 0.00 | ARD |
| Dominant vessel multiple type | Absent, Radial multiples, Clusters | 0.00 | ARD |

Model selection of ancestral state reconstruction: polymorphic traits

Model selection of polymorphic states for stochastic mapping. Model with the lowest AIC score was selected (in bold). ERor = equal rates model, characters ordered; ERun = equal rates model, unordered; ARDor = all rates different model, ordered; ARDun = all rates different model, unordered; SYMor = symmetric backward & forward rates model, ordered, SYMun = symmetric backward & forward rates model, unordered; TRAor = transient model, ordered; TRAun = transient model, unordered.

| **Trait** | **States** | **AIC score** | | | | | | | |
| --- | --- | --- | --- | --- | --- | --- | --- | --- | --- |
|  |  | ERor | ERun | ARDor | ARDun | SYMor | SYMun | TRAor | TRAun |
| Ray width | Uniseriate, Multiseriate, Uniseriate+Multiseriate | 324.9920 | 324.9920 | 263.3699 | **263.3699** | 271.3612 | 271.3612 | 276.2733 | 276.2733 |
| Parenchyma | Absent, Apotracheal, Paratracheal, Apotracheal+Paratracheal | 468.3920 | 509.3723 | 367.6084 | **357.1741** | 459.2559 | 410.4040 | 402.1743 | 387.3993 |
| Growth form | Erect, Climbing, Erect+Climbing | 192.9507 | 192.9507 | 134.2231 | **134.2231** | 183.9799 | 183.9799 | 136.0270 | 136.0270 |
| Prismatic crystal position | Absent, Axial parenchyma, Rays, Axial parenchyma+Rays | 473.9989 | 574.0976 | **368.9822** | 380.5649 | 901.5046 | 457.3393 | 404.4614 | 411.5077 |
| Ground tissue type | Tracheids, Fibre-tracheids, Libriform fibres, Tracheids+Fibre-tracheids, Tracheids+Libirform fibres, Tracheids+Fibre-tracheids+Libriform fibres, Fibre-Tracheids+Libriform fibres | ERROR | 423.2715 | ERROR | **355.8938** | ERROR | 366.6141 | ERROR | 378.1238 |
