# Supplementary material for: Apocynaceae wood evolution matches key morphological innovations
Source: Am J Bot. 2024 Nov 22;111(11):e16436. doi: 10.1002/ajb2.16436 (PMC11584039; doi:10.1002/ajb2.16436)
Supplement: Supplementary file 5 — Appendix S5. Visualization of ancestral state reconstructions with species names as tip labels and reconstructions not included in the main text. [file AJB2-111-e16436-s005.pdf]

Beckers et al.—American Journal of Botany 2024—Appendix S5. visualization of ancestral state reconstructions with species names as tip labels and reconstructions not included in the main text.

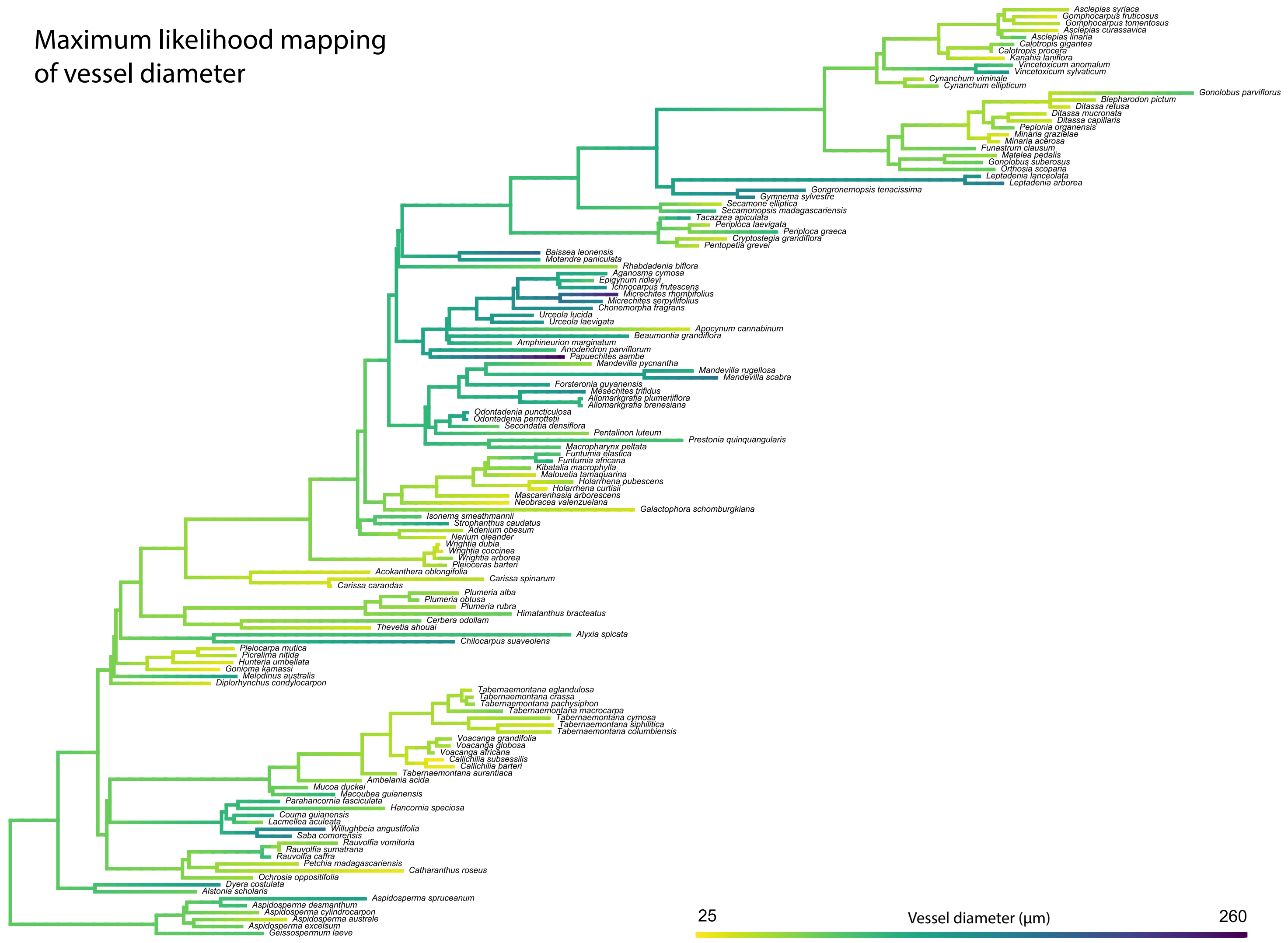

Maximum likelihood mapping of vessel diameter
Asclepias syriaca
Gomphocarpus fruticosus
Gomphocarpus tomentosus
Asclepias curassavica
Asclepias linaria
Calotropis gigantea
Calotropis procera
Kanahia laniflora
Vincetoxicum anomalum
Vincetoxicum sylvaticum
Cynanchum viminale
Cynanchum ellipticum
Gonolobus parviflorus
Blepharodon pictum
Ditassa retusa
Ditassa mucronata
Ditassa capillaris
Peplonia organensis
Minaria grazielae
Minaria acerosa
Funastrum clausum
Matelea pedalis
Gonolobus suberosus
Orthosia scoparia
Leptadenia lanceolata
Leptadenia arborea
Gongronemopsis tenacissima
Gymnema sylvestre
Secamone elliptica
Secamonopsis madagascariensis
Tacazzea apiculata
Periploca laevigata
Periploca graeca
Cryptostegia grandiflora
Pentopetia grevei
Baissea leonensis
Motandra paniculata
Rhabdadenia biflora
Aganosma cymosa
Epigynum ridleyi
Ichnocarpus frutescens
Micrechites rhombifolius
Micrechites serpyllifolius
Chonemorpha fragrans
Urceola lucida
Urceola laevigata
Apocynum cannabinum
Beaumontia grandiflora
Amphineurion marginatum
Anodendron parviflorum
Papuechites aambe
Mandevilla pycnantha
Mandevilla rugellosa
Mandevilla scabra
Forsteronia guyanensis
Mesechites trifidus
Allomarkgrafia plumeriiflora
Allomarkgrafia brenesiana
Odontadenia puncticulosa
Odontadenia perrottetii
Secondatia densiflora
Pentalinon luteum
Prestonia quinquangularis
Macropharynx peltata
Funtumia elastica
Funtumia africana
Kibatalia macrophylla
Malouetia tamaquarina
Holarrhena pubescens
Holarrhena curtisii
Mascarenhasia arborescens
Neobracea valenzuelana
Galactophora schomburgkiana
Isonema smeathmannii
Strophanthus caudatus
Adenium obesum
Nerium oleander
Wrightia dubia
Wrightia coccinea
Wrightia arborea
Pleioceras barteri
Acokanthera oblongifolia
Carissa spinarum
Carissa carandas
Plumeria alba
Plumeria obtusa
Plumeria rubra
Himatanthus bracteatus
Cerbera odollam
Thevetia ahouai
Alyxia spicata
Chilocarpus suaveolens
Pleiocarpa mutica
Picralima nitida
Hunteria umbellata
Gonioma kamassi
Melodinus australis
Diplorhynchus condylocarpon
Tabernaemontana eglandulosa
Tabernaemontana crassa
Tabernaemontana pachysiphon
Tabernaemontana macrocarpa
Tabernaemontana cymosa
Tabernaemontana siphilitica
Tabernaemontana columbiensis
Voacanga grandifolia
Voacanga globosa
Voacanga africana
Callichilia subsessilis
Callichilia barteri
Tabernaemontana aurantiaca
Ambelania acida
Mucoa duckei
Macoubea guianensis
Parahancornia fasciculata
Hancornia speciosa
Couma guianensis
Lacmellea aculeata
Willughbeia angustifolia
Saba comorensis
Rauvolfia vomitoria
Rauvolfia sumatrana
Rauvolfia caffra
Petchia madagascariensis
Catharanthus roseus
Ochrosia oppositifolia
Dyera costulata
Alstonia scholaris
Aspidosperma spruceanum
Aspidosperma desmanthum
Aspidosperma cylindrocarpon
Aspidosperma australe
Aspidosperma excelsum
Geissospermum laeve
25
Vessel diameter (µm)
260

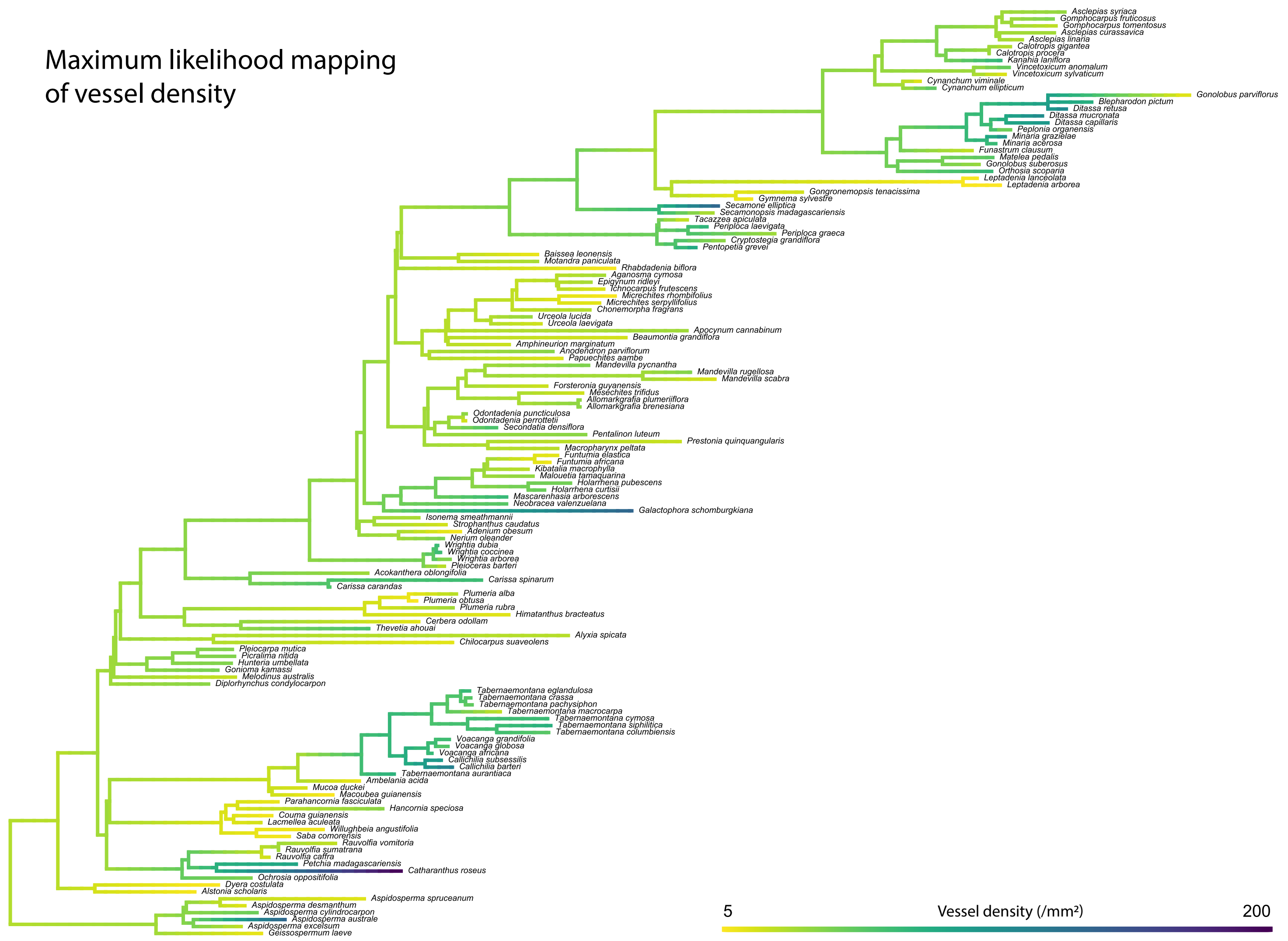

Maximum likelihood mapping of vessel density
Asclepias syriaca
Gomphocarpus fruticosus
Gomphocarpus tomentosus
Asclepias curassavica
Asclepias linaria
Calotropis gigantea
Calotropis procera
Kanahia laniflora
Vincetoxicum anomalum
Vincetoxicum sylvaticum
Cynanchum viminale
Cynanchum ellipticum
Gonolobus parviflorus
Blepharodon pictum
Ditassa retusa
Ditassa mucronata
Ditassa capillaris
Peplonia organensis
Minaria grazielae
Minaria acerosa
Funastrum clausum
Matelea pedalis
Gonolobus suberosus
Orthosia scoparia
Leptadenia lanceolata
Leptadenia arborea
Gongronemopsis tenacissima
Gymnema sylvestre
Secamone elliptica
Secamonopsis madagascariensis
Tacazzea apiculata
Periploca laevigata
Periploca graeca
Cryptostegia grandiflora
Pentopetia grevei
Baissea leonensis
Motandra paniculata
Rhabdadenia biflora
Aganosma cymosa
Epigynum ridleyi
Ichnocarpus frutescens
Micrechites rhombifolius
Micrechites serpyllifolius
Chonemorpha fragrans
Urceola lucida
Urceola laevigata
Apocynum cannabinum
Beaumontia grandiflora
Amphineurion marginatum
Anodendron parviflorum
Papuechites aambe
Mandevilla pycnantha
Mandevilla rugellosa
Mandevilla scabra
Forsteronia guyanensis
Mesechites trifidus
Allomarkgrafia plumeriiflora
Allomarkgrafia brenesiana
Odontadenia puncticulosa
Odontadenia perrottetii
Secondatia densiflora
Pentalinon luteum
Prestonia quinquangularis
Macropharynx peltata
Funtumia elastica
Funtumia africana
Kibatalia macrophylla
Malouetia tamaquarina
Holarrhena pubescens
Holarrhena curtisii
Mascarenhasia arborescens
Neobracea valenzuelana
Galactophora schomburgkiana
Isonema smeathmannii
Strophanthus caudatus
Adenium obesum
Nerium oleander
Wrightia dubia
Wrightia coccinea
Wrightia arborea
Pleioceras barteri
Acokanthera oblongifolia
Carissa spinarum
Carissa carandas
Plumeria alba
Plumeria obtusa
Plumeria rubra
Himatanthus bracteatus
Cerbera odollam
Thevetia ahouai
Alyxia spicata
Chilocarpus suaveolens
Pleiocarpa mutica
Picralima nitida
Hunteria umbellata
Gonioma kamassi
Melodinus australis
Diplorhynchus condylocarpon
Tabernaemontana eglandulosa
Tabernaemontana crassa
Tabernaemontana pachysiphon
Tabernaemontana macrocarpa
Tabernaemontana cymosa
Tabernaemontana siphilitica
Tabernaemontana columbiensis
Voacanga grandifolia
Voacanga globosa
Voacanga africana
Callichilia subsessilis
Callichilia barteri
Tabernaemontana aurantiaca
Ambelania acida
Mucoa duckei
Macoubea guianensis
Parahancornia fasciculata
Hancornia speciosa
Couma guianensis
Lacmellea aculeata
Willughbeia angustifolia
Saba comorensis
Rauvolfia vomitoria
Rauvolfia sumatrana
Rauvolfia caffra
Petchia madagascariensis
Catharanthus roseus
Ochrosia oppositifolia
Dyera costulata
Alstonia scholaris
Aspidosperma spruceanum
Aspidosperma desmanthum
Aspidosperma cylindrocarpon
Aspidosperma australe
Aspidosperma excelsum
Geissospermum laeve
5
Vessel density (/mm²)
200

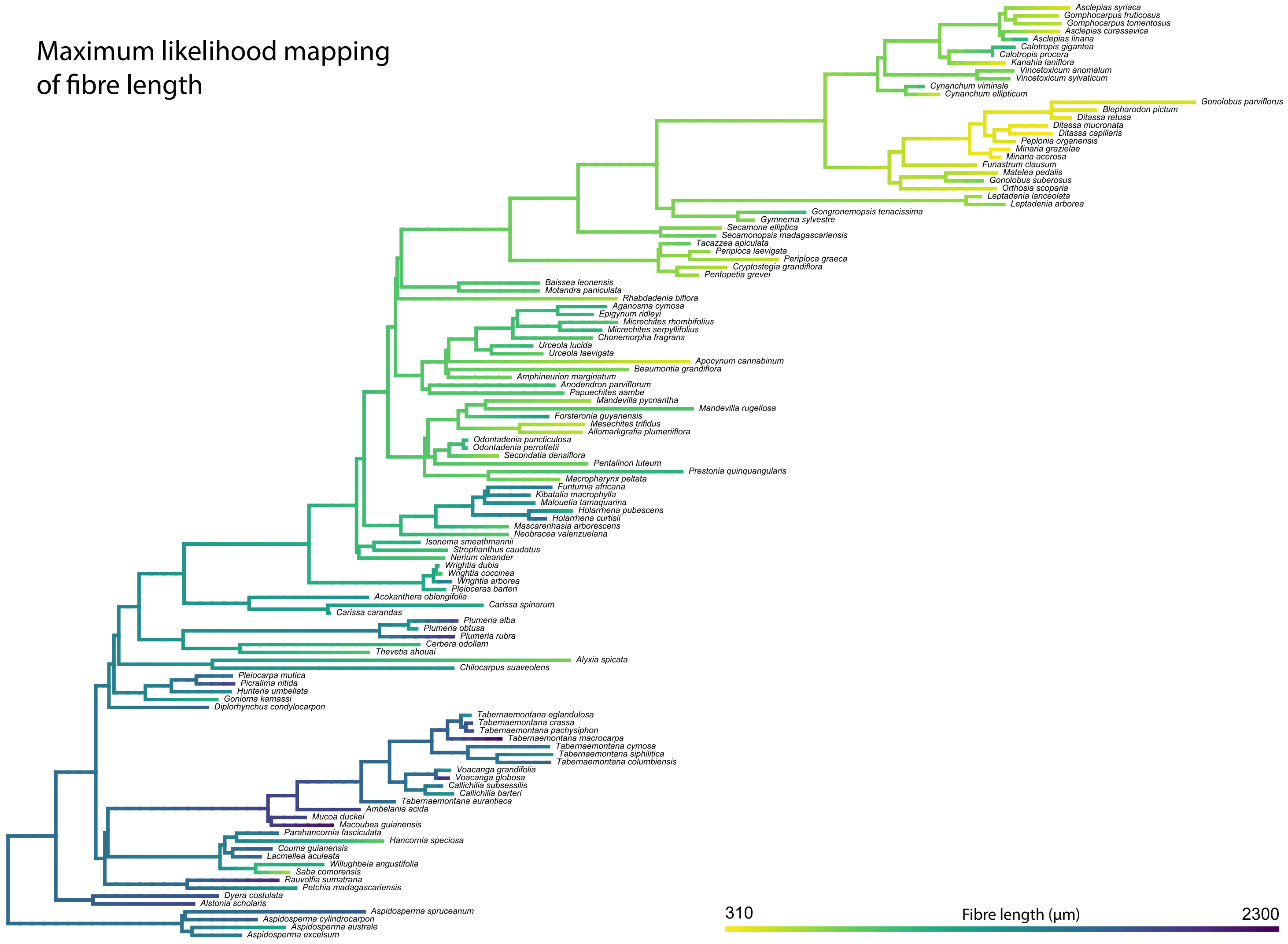

Maximum likelihood mapping of fibre length
Asclepias syriaca
Gomphocarpus fruticosus
Gomphocarpus tomentosus
Asclepias curassavica
Asclepias linaria
Calotropis gigantea
Calotropis procera
Kanahia laniflora
Vincetoxicum anomalum
Vincetoxicum sylvaticum
Cynanchum viminale
Cynanchum ellipticum
Gonolobus parviflorus
Blepharodon pictum
Ditassa retusa
Ditassa mucronata
Ditassa capillaris
Peplonia organensis
Minaria grazielae
Minaria acerosa
Funastrum clausum
Matelea pedalis
Gonolobus suberosus
Orthosia scoparia
Leptadenia lanceolata
Leptadenia arborea
Gongronemopsis tenacissima
Gymnema sylvestre
Secamone elliptica
Secamonopsis madagascariensis
Tacazzea apiculata
Periploca laevigata
Periploca graeca
Cryptostegia grandiflora
Pentopetia grevei
Baissea leonensis
Motandra paniculata
Rhabdadenia biflora
Aganosma cymosa
Epigynum ridleyi
Micrechites rhombifolius
Micrechites serpyllifolius
Chonemorpha fragrans
Urceola lucida
Urceola laevigata
Apocynum cannabinum
Beaumontia grandiflora
Amphineurion marginatum
Anodendron parviflorum
Papuechites aambe
Mandevilla pycnantha
Mandevilla rugellosa
Forsteronia guyanensis
Mesechites trifidus
Allomarkgrafia plumeriiflora
Odontadenia puncticulosa
Odontadenia perrottetii
Secondatia densiflora
Pentalinon luteum
Prestonia quinquangularis
Macropharynx peltata
Funtumia africana
Kibatalia macrophylla
Malouetia tamaquarina
Holarrhena pubescens
Holarrhena curtisii
Mascarenhasia arborescens
Neobracea valenzuelana
Isonema smeathmannii
Strophanthus caudatus
Nerium oleander
Wrightia dubia
Wrightia coccinea
Wrightia arborea
Pleioceras barteri
Acokanthera oblongifolia
Carissa spinarum
Carissa carandas
Plumeria alba
Plumeria obtusa
Plumeria rubra
Cerbera odollam
Thevetia ahouai
Alyxia spicata
Chilocarpus suaveolens
Pleiocarpa mutica
Picralima nitida
Hunteria umbellata
Gonioma kamassi
Diplorhynchus condylocarpon
Tabernaemontana eglandulosa
Tabernaemontana crassa
Tabernaemontana pachysiphon
Tabernaemontana macrocarpa
Tabernaemontana cymosa
Tabernaemontana siphilitica
Tabernaemontana columbiensis
Voacanga grandifolia
Voacanga globosa
Callichilia subsessilis
Callichilia barteri
Tabernaemontana aurantiaca
Ambelania acida
Mucoa duckei
Macoubea guianensis
Parahancornia fasciculata
Hancornia speciosa
Couma guianensis
Lacmellea aculeata
Willughbeia angustifolia
Saba comorensis
Rauvolfia sumatrana
Petchia madagascariensis
Dyera costulata
Alstonia scholaris
Aspidosperma spruceanum
Aspidosperma cylindrocarpon
Aspidosperma australe
Aspidosperma excelsum
310
Fibre length (μm)
2300

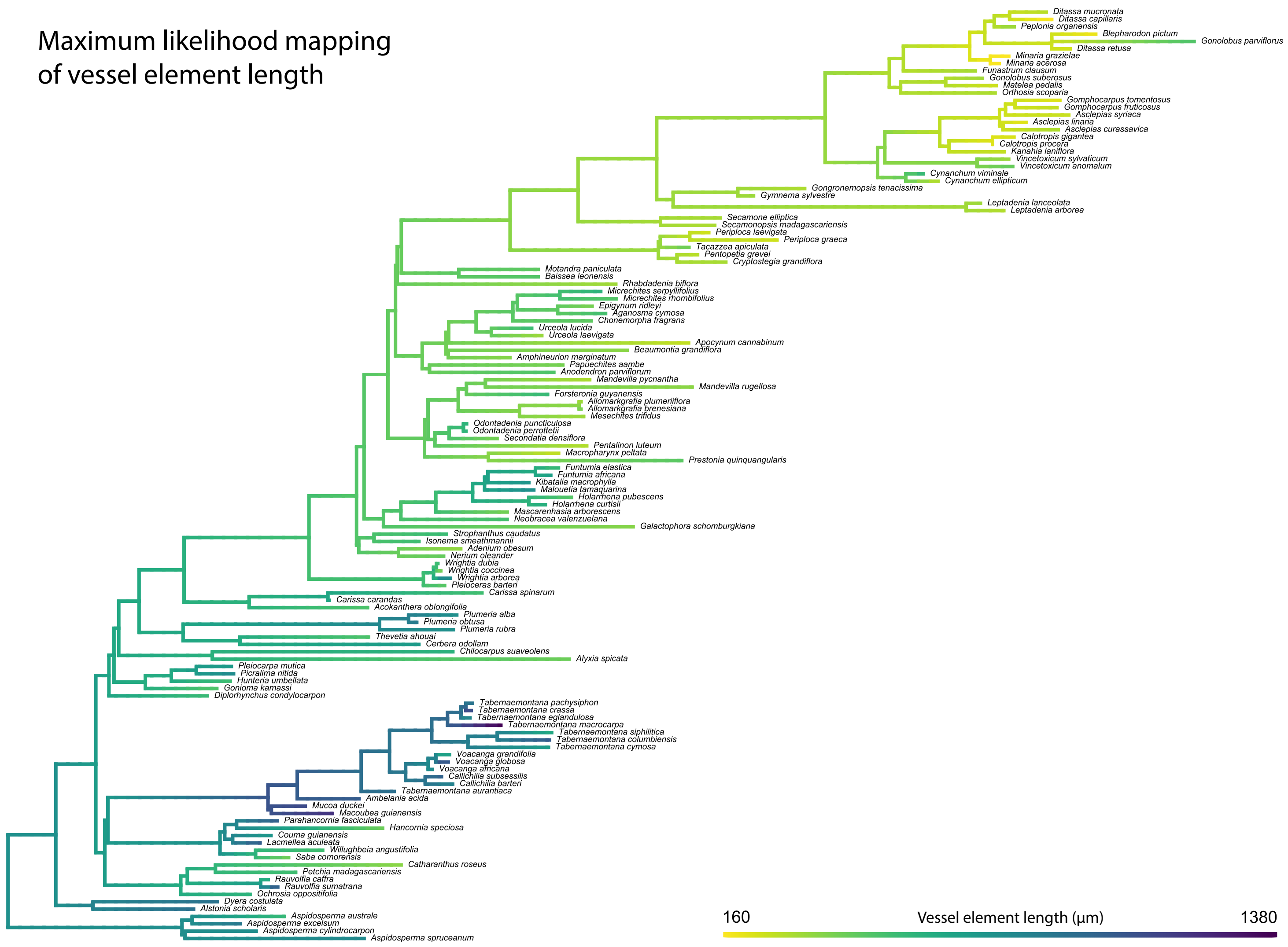

Maximum likelihood mapping of vessel element length
Ditassa mucronata
Ditassa capillaris
Peplonia organensis
Blepharodon pictum
Gonolobus parviflorus
Ditassa retusa
Minaria grazielae
Minaria acerosa
Funastrum clausum
Gonolobus suberosus
Matelea pedalis
Orthosia scoparia
Gomphocarpus tomentosus
Gomphocarpus fruticosus
Asclepias syriaca
Asclepias linaria
Asclepias curassavica
Calotropis gigantea
Calotropis procera
Kanahia laniflora
Vincetoxicum sylvaticum
Vincetoxicum anomalum
Cynanchum viminale
Cynanchum ellipticum
Gongronemopsis tenacissima
Gymnema sylvestre
Leptadenia lanceolata
Leptadenia arborea
Secamone elliptica
Secamonopsis madagascariensis
Periploca laevigata
Periploca graeca
Tacazzea apiculata
Pentopetia grevei
Cryptostegia grandiflora
Motandra paniculata
Baissea leonensis
Rhabdadenia biflora
Micrechites serpyllifolius
Micrechites rhombifolius
Epigynum ridleyi
Aganosma cymosa
Chonemorpha fragrans
Urceola lucida
Urceola laevigata
Apocynum cannabinum
Beaumontia grandiflora
Amphineurion marginatum
Papuechites aambe
Anodendron parviflorum
Mandevilla pycnantha
Mandevilla rugellosa
Forsteronia guyanensis
Allomarkgrafia plumeriiflora
Allomarkgrafia brenesiana
Mesechites trifidus
Odontadenia puncticulosa
Odontadenia perrottetii
Secondatia densiflora
Pentalinon luteum
Macropharynx peltata
Prestonia quinquangularis
Funtumia elastica
Funtumia africana
Kibatalia macrophylla
Malouetia tamaquarina
Holarrhena pubescens
Holarrhena curtisii
Mascarenhasia arborescens
Neobracea valenzuelana
Galactophora schomburgkiana
Strophanthus caudatus
Isonema smeathmannii
Adenium obesum
Nerium oleander
Wrightia dubia
Wrightia coccinea
Wrightia arborea
Pleioceras barteri
Carissa spinarum
Carissa carandas
Acokanthera oblongifolia
Plumeria alba
Plumeria obtusa
Plumeria rubra
Thevetia ahouai
Cerbera odollam
Chilocarpus suaveolens
Alyxia spicata
Pleiocarpa mutica
Picralima nitida
Hunteria umbellata
Gonioma kamassi
Diplorhynchus condylocarpon
Tabernaemontana pachysiphon
Tabernaemontana crassa
Tabernaemontana eglandulosa
Tabernaemontana macrocarpa
Tabernaemontana siphilitica
Tabernaemontana columbiensis
Tabernaemontana cymosa
Voacanga grandifolia
Voacanga globosa
Voacanga africana
Callichilia subsessilis
Callichilia barteri
Tabernaemontana aurantiaca
Ambelania acida
Mucoa duckei
Macoubea guianensis
Parahancornia fasciculata
Hancornia speciosa
Couma guianensis
Lacmellea aculeata
Willughbeia angustifolia
Saba comorensis
Catharanthus roseus
Petchia madagascariensis
Rauvolfia caffra
Rauvolfia sumatrana
Ochrosia oppositifolia
Dyera costulata
Alstonia scholaris
Aspidosperma australe
Aspidosperma excelsum
Aspidosperma cylindrocarpon
Aspidosperma spruceanum
160
Vessel element length (μm)
1380

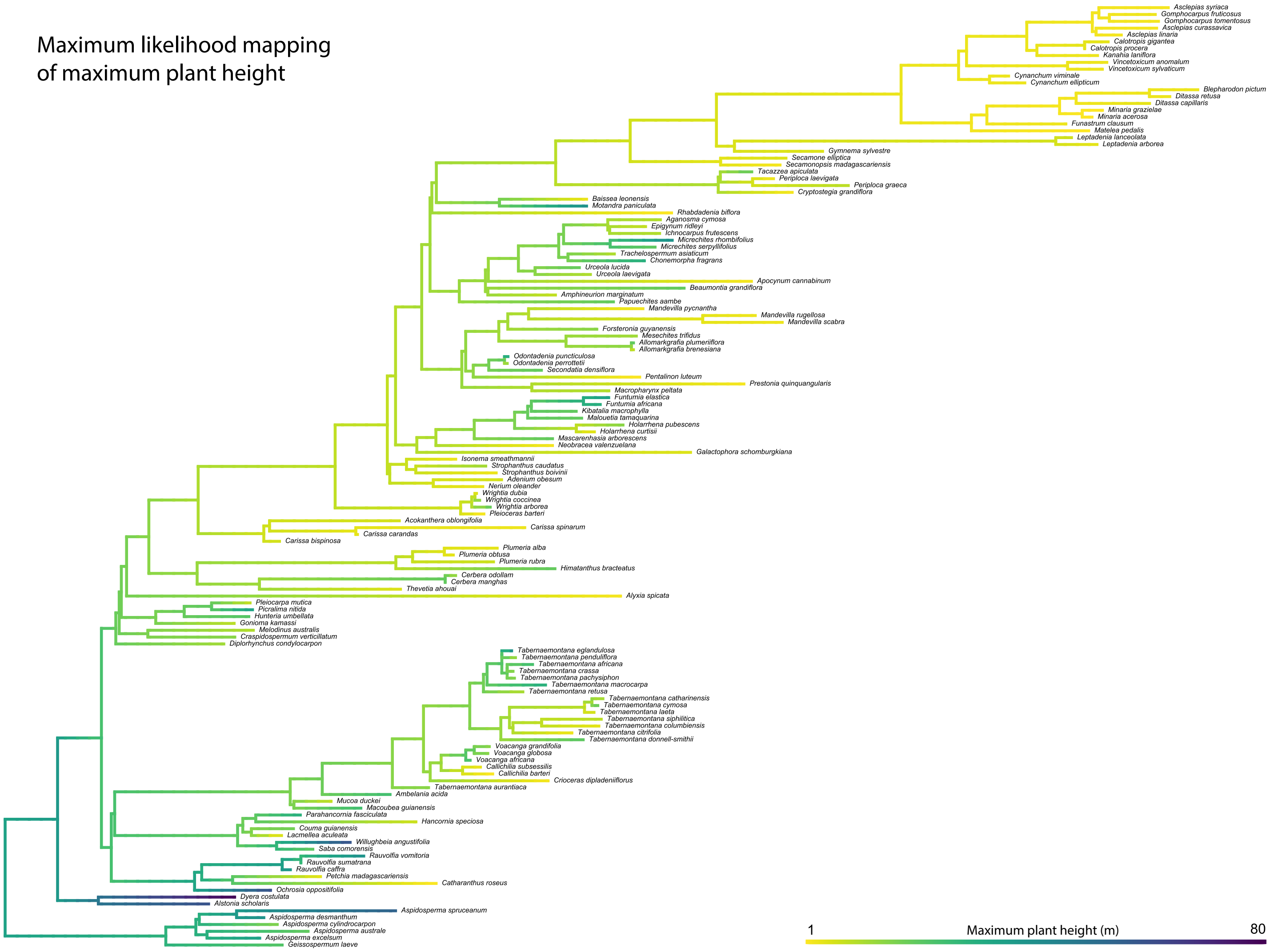
Maximum likelihood mapping
of maximum plant height
Asclepias syriaca
Gomphocarpus fruticosus
Gomphocarpus tomentosus
Asclepias curassavica
Asclepias linaria
Calotropis gigantea
Calotropis procera
Kanahia laniflora
Vincetoxicum anomalum
Vincetoxicum sylvaticum
Cynanchum viminale
Cynanchum ellipticum
Blepharodon pictum
Ditassa retusa
Ditassa capillaris
Minaria grazielae
Minaria acerosa
Funastrum clausum
Matelea pedalis
Leptadenia lanceolata
Leptadenia arborea
Gymnema sylvestre
Secamone elliptica
Secamonopsis madagascariensis
Tacazzea apiculata
Periploca laevigata
Periploca graeca
Cryptostegia grandiflora
Baissea leonensis
Motandra paniculata
Rhabdadenia biflora
Aganosma cymosa
Epigynum ridleyi
Ichnocarpus frutescens
Micrechites rhombifolius
Micrechites serpyllifolius
Trachelospermum asiaticum
Chonemorpha fragrans
Urceola lucida
Urceola laevigata
Apocynum cannabinum
Beaumontia grandiflora
Amphineurion marginatum
Papuechites aambe
Mandevilla pycnantha
Mandevilla rugellosa
Mandevilla scabra
Forsteronia guyanensis
Mesechites trifidus
Allomarkgrafia plumeriiflora
Allomarkgrafia brenesiana
Odontadenia puncticulosa
Odontadenia perrottetii
Secondatia densiflora
Pentalinon luteum
Prestonia quinquangularis
Macropharynx peltata
Funtumia elastica
Funtumia africana
Kibatalia macrophylla
Malouetia tamaquarina
Holarrhena pubescens
Holarrhena curtisii
Mascarenhasia arborescens
Neobracea valenzuelana
Galactophora schomburgkiana
Isonema smeathmannii
Strophanthus caudatus
Strophanthus boivinii
Adenium obesum
Nerium oleander
Wrightia dubia
Wrightia coccinea
Wrightia arborea
Pleioceras barteri
Acokanthera oblongifolia
Carissa spinarum
Carissa carandas
Carissa bispinosa
Plumeria alba
Plumeria obtusa
Plumeria rubra
Himatanthus bracteatus
Cerbera odollam
Cerbera manghas
Thevetia ahouai
Alyxia spicata
Pleiocarpa mutica
Picralima nitida
Hunteria umbellata
Gonioma kamassi
Melodinus australis
Craspidospermum verticillatum
Diplorhynchus condylocarpon
Tabernaemontana eglandulosa
Tabernaemontana penduliflora
Tabernaemontana africana
Tabernaemontana crassa
Tabernaemontana pachysiphon
Tabernaemontana macrocarpa
Tabernaemontana retusa
Tabernaemontana catharinensis
Tabernaemontana cymosa
Tabernaemontana laeta
Tabernaemontana siphilitica
Tabernaemontana columbiensis
Tabernaemontana citrifolia
Tabernaemontana donnell-smithii
Voacanga grandifolia
Voacanga globosa
Voacanga africana
Callichilia subsessilis
Callichilia barteri
Crioceras dipladeniiflorus
Tabernaemontana aurantiaca
Ambelania acida
Mucoa duckei
Macoubea guianensis
Parahancornia fasciculata
Hancornia speciosa
Couma guianensis
Lacmellea aculeata
Willughbeia angustifolia
Saba comorensis
Rauvolfia vomitoria
Rauvolfia sumatrana
Rauvolfia caffra
Petchia madagascariensis
Catharanthus roseus
Ochrosia oppositifolia
Dyera costulata
Alstonia scholaris
Aspidosperma spruceanum
Aspidosperma desmanthum
Aspidosperma cylindrocarpon
Aspidosperma australe
Aspidosperma excelsum
Geissospermum laeve
1
Maximum plant height (m)
80

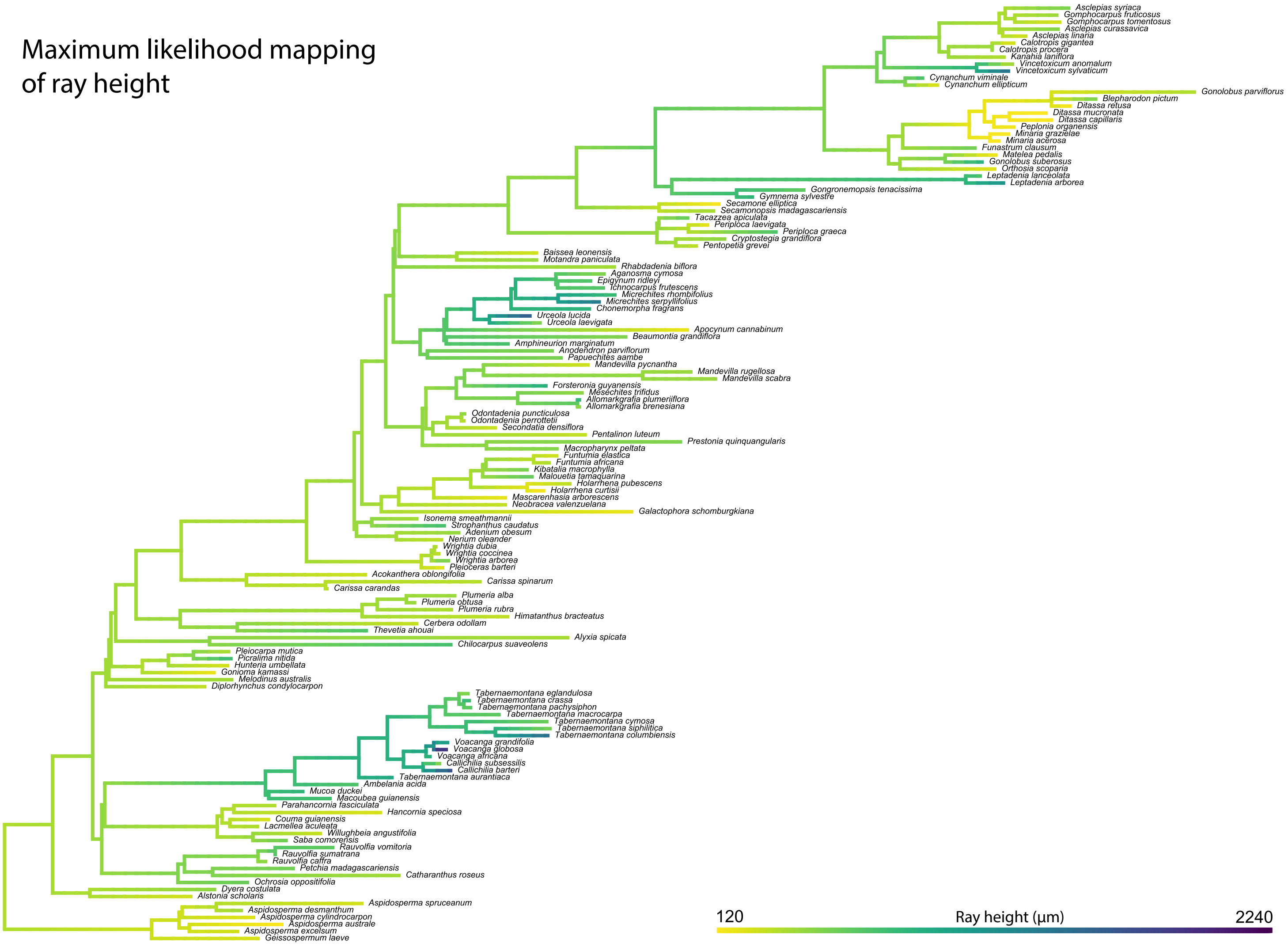
Maximum likelihood mapping
of ray height
Asclepias syriaca
Gomphocarpus fruticosus
Gomphocarpus tomentosus
Asclepias curassavica
Asclepias linaria
Calotropis gigantea
Calotropis procera
Kanahia laniflora
Vincetoxicum anomalum
Vincetoxicum sylvaticum
Cynanchum viminale
Cynanchum ellipticum
Gonolobus parviflorus
Blepharodon pictum
Ditassa retusa
Ditassa mucronata
Ditassa capillaris
Peplonia organensis
Minaria grazielae
Minaria acerosa
Funastrum clausum
Matelea pedalis
Gonolobus suberosus
Orthosia scoparia
Leptadenia lanceolata
Leptadenia arborea
Gongronemopsis tenacissima
Gymnema sylvestre
Secamone elliptica
Secamonopsis madagascariensis
Tacazzea apiculata
Periploca laevigata
Periploca graeca
Cryptostegia grandiflora
Pentopetia grevei
Baissea leonensis
Motandra paniculata
Rhabdadenia biflora
Aganosma cymosa
Epigynum ridleyi
Ichnocarpus frutescens
Micrechites rhombifolius
Micrechites serpyllifolius
Chonemorpha fragrans
Urceola lucida
Urceola laevigata
Apocynum cannabinum
Beaumontia grandiflora
Amphineurion marginatum
Anodendron parviflorum
Papuechites aambe
Mandevilla pycnantha
Mandevilla rugellosa
Mandevilla scabra
Forsteronia guyanensis
Mesechites trifidus
Allomarkgrafia plumeriiflora
Allomarkgrafia brenesiana
Odontadenia puncticulosa
Odontadenia perrottetii
Secondatia densiflora
Pentalinon luteum
Prestonia quinquangularis
Macropharynx peltata
Funtumia elastica
Funtumia africana
Kibatalia macrophylla
Malouetia tamaquarina
Holarrhena pubescens
Holarrhena curtisii
Mascarenhasia arborescens
Neobracea valenzuelana
Galactophora schomburgkiana
Isonema smeathmannii
Strophanthus caudatus
Adenium obesum
Nerium oleander
Wrightia dubia
Wrightia coccinea
Wrightia arborea
Pleioceras barteri
Acokanthera oblongifolia
Carissa spinarum
Carissa carandas
Plumeria alba
Plumeria obtusa
Plumeria rubra
Himatanthus bracteatus
Cerbera odollam
Thevetia ahouai
Alyxia spicata
Chilocarpus suaveolens
Pleiocarpa mutica
Picralima nitida
Hunteria umbellata
Gonioma kamassi
Melodinus australis
Diplorhynchus condylocarpon
Tabernaemontana eglandulosa
Tabernaemontana crassa
Tabernaemontana pachysiphon
Tabernaemontana macrocarpa
Tabernaemontana cymosa
Tabernaemontana siphilitica
Tabernaemontana columbiensis
Voacanga grandifolia
Voacanga globosa
Voacanga africana
Callichilia subsessilis
Callichilia barteri
Tabernaemontana aurantiaca
Ambelania acida
Mucoa duckei
Macoubea guianensis
Parahancornia fasciculata
Hancornia speciosa
Couma guianensis
Lacmellea aculeata
Willughbeia angustifolia
Saba comorensis
Rauvolfia vomitoria
Rauvolfia sumatrana
Rauvolfia caffra
Petchia madagascariensis
Catharanthus roseus
Ochrosia oppositifolia
Dyera costulata
Alstonia scholaris
Aspidosperma spruceanum
Aspidosperma desmanthum
Aspidosperma cylindrocarpon
Aspidosperma australe
Aspidosperma excelsum
Geissospermum laeve
120
Ray height (µm)
2240

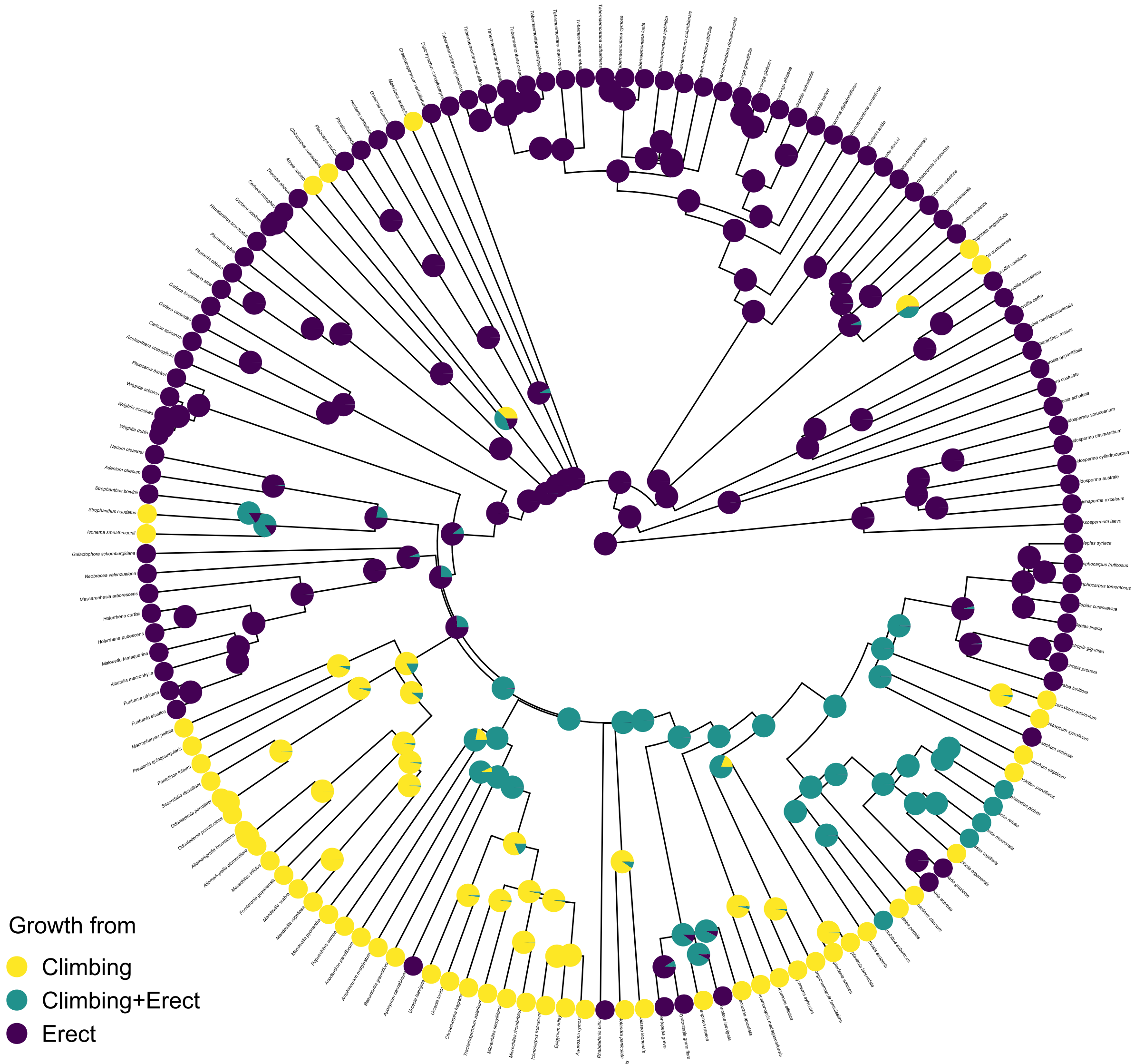
Growth from
Climbing
Climbing+Erect
Erect

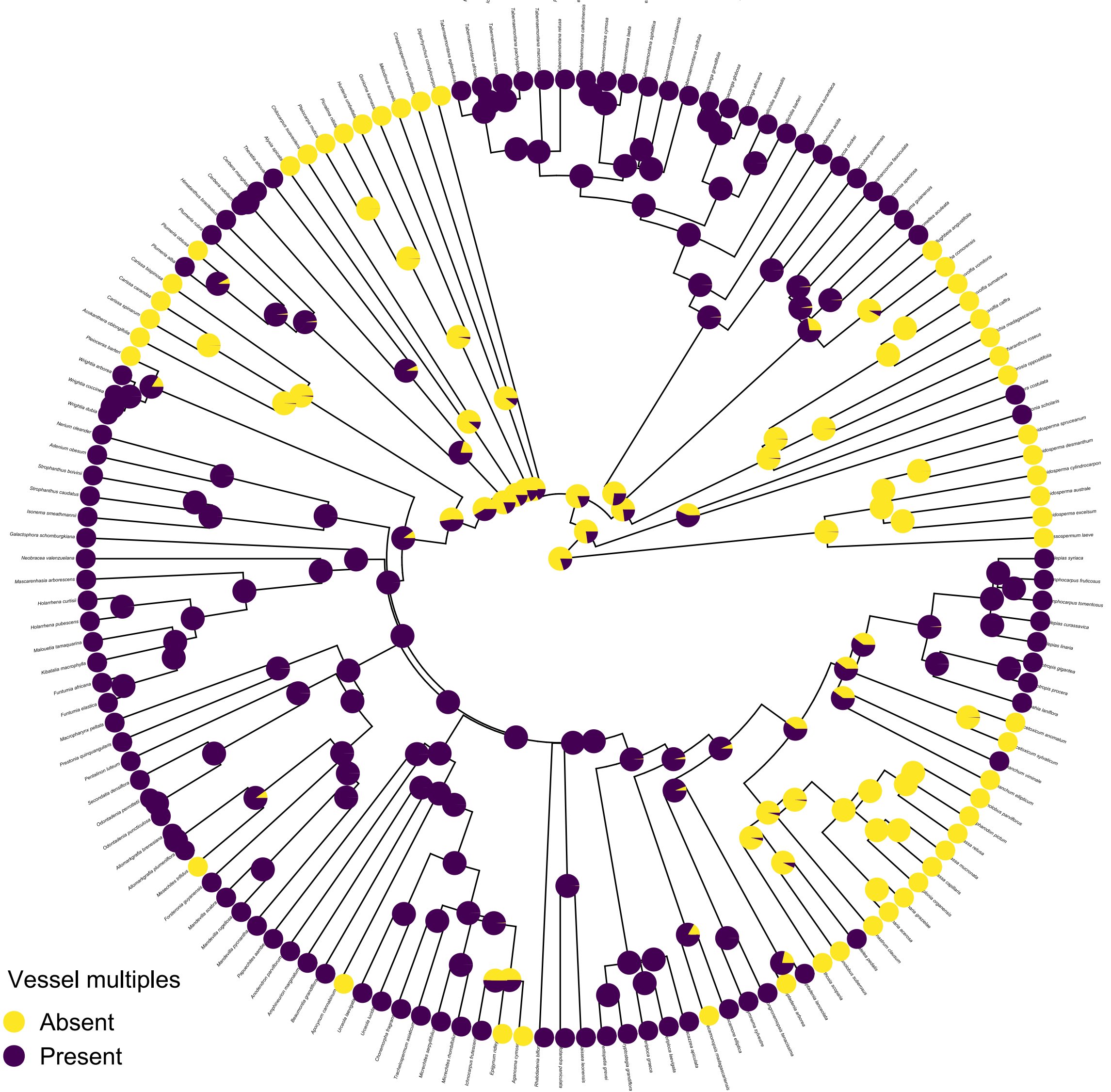
Vessel multiples
Absent
Present

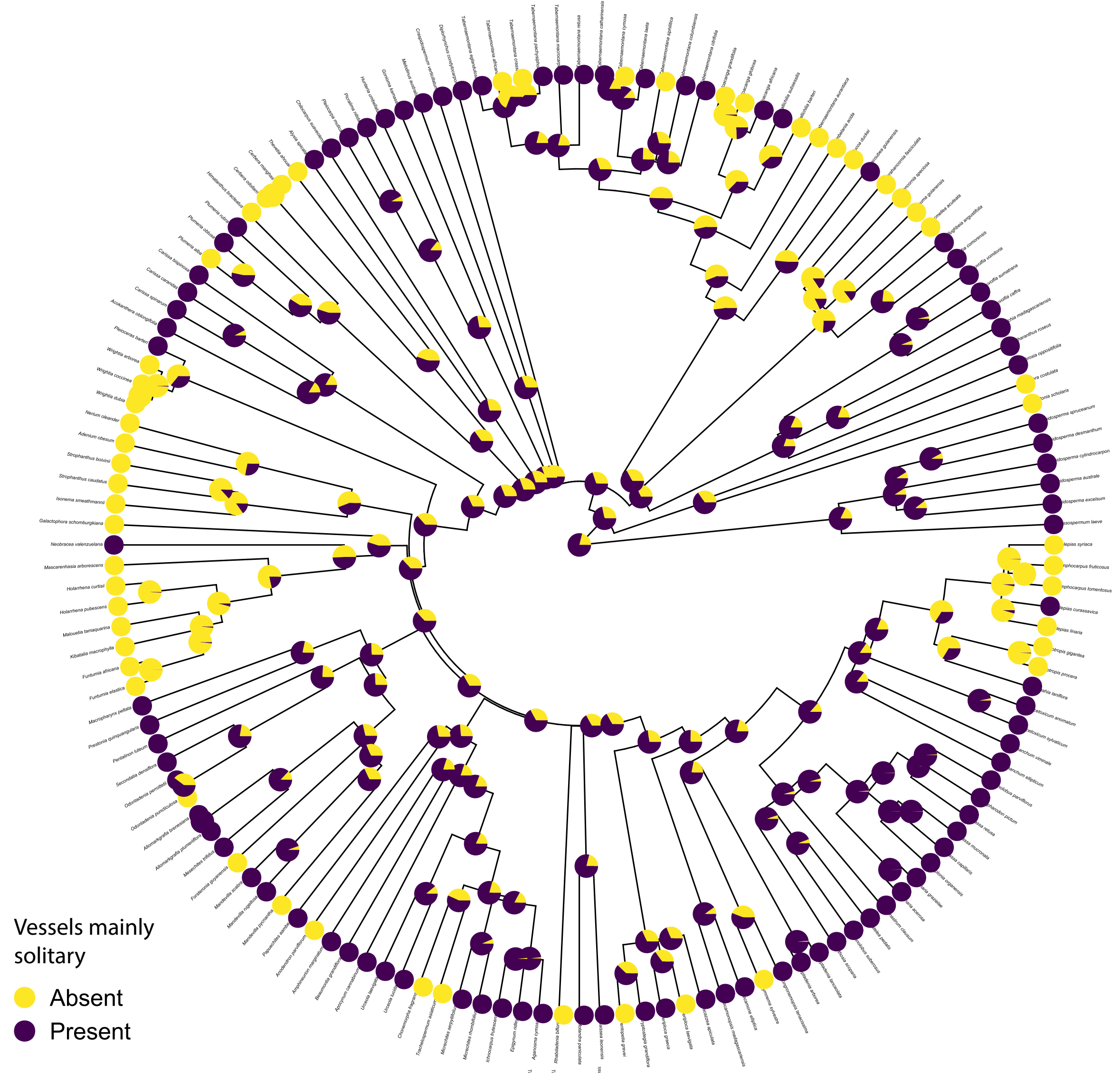
Vessels mainly solitary
Absent
Present

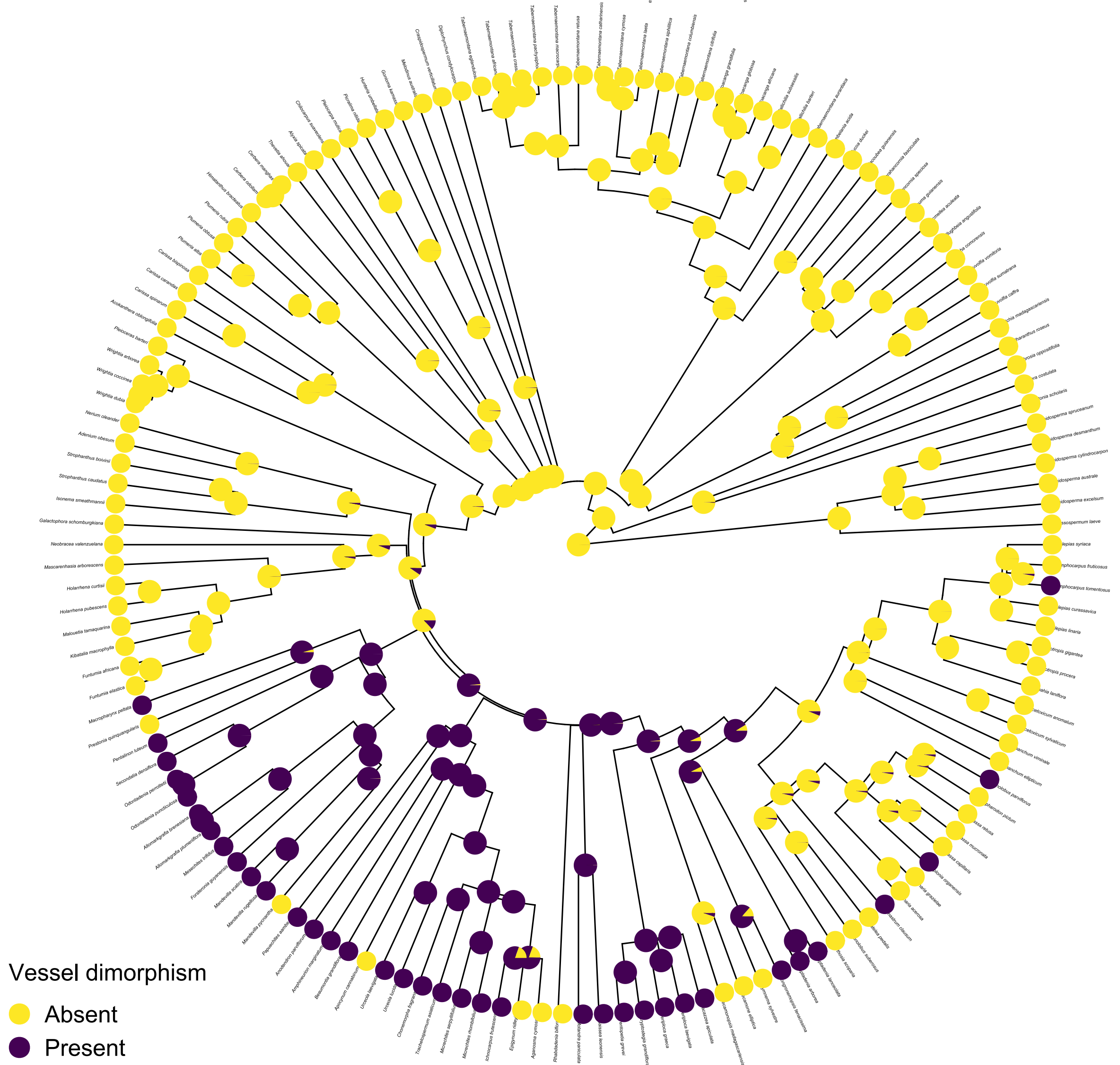
Vessel dimorphism
Absent
Present

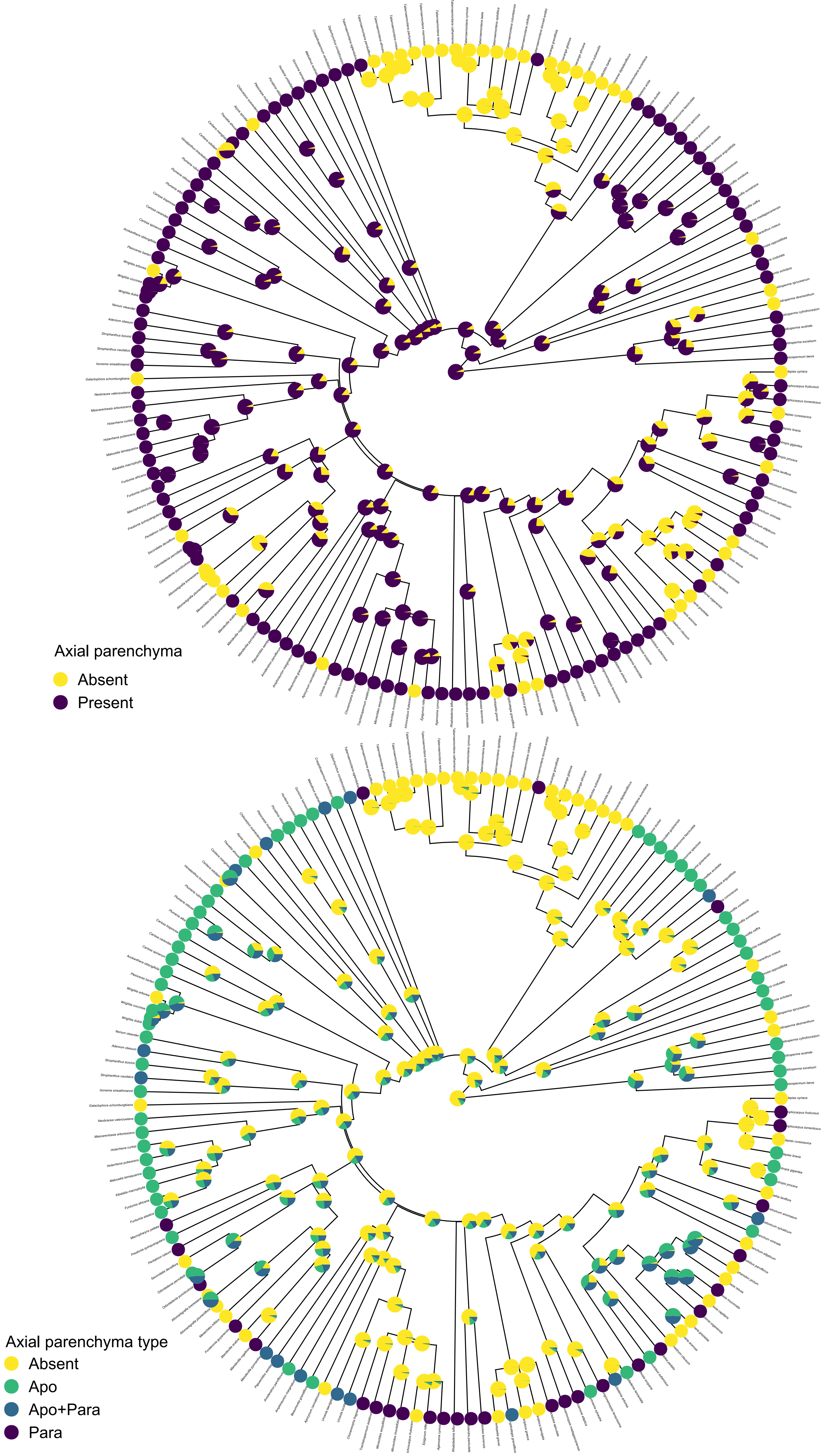

Axial parenchyma
Absent
Present
Axial parenchyma type
Absent
Apo
Apo+Para
Para

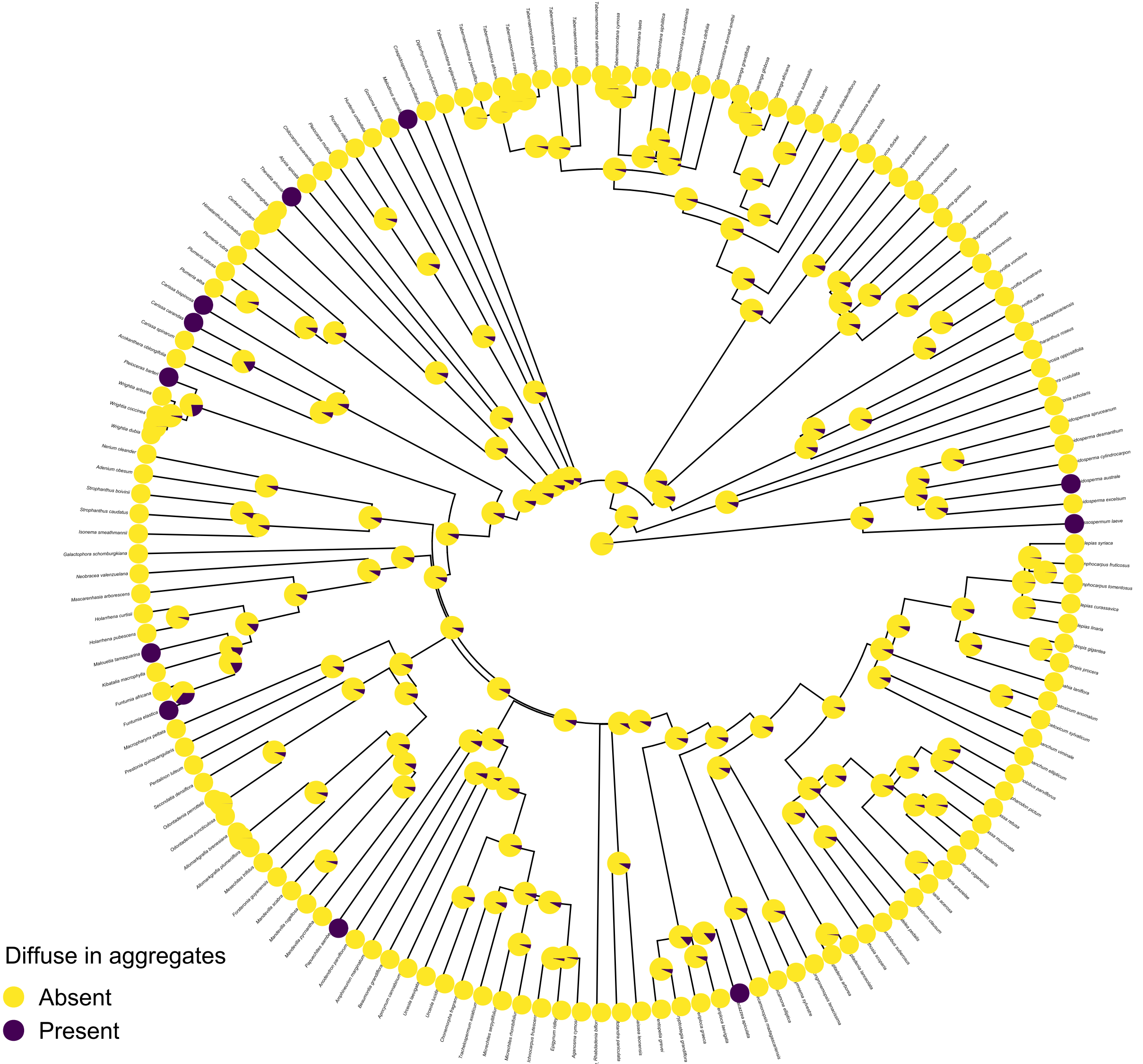
Diffuse in aggregates
Absent
Present

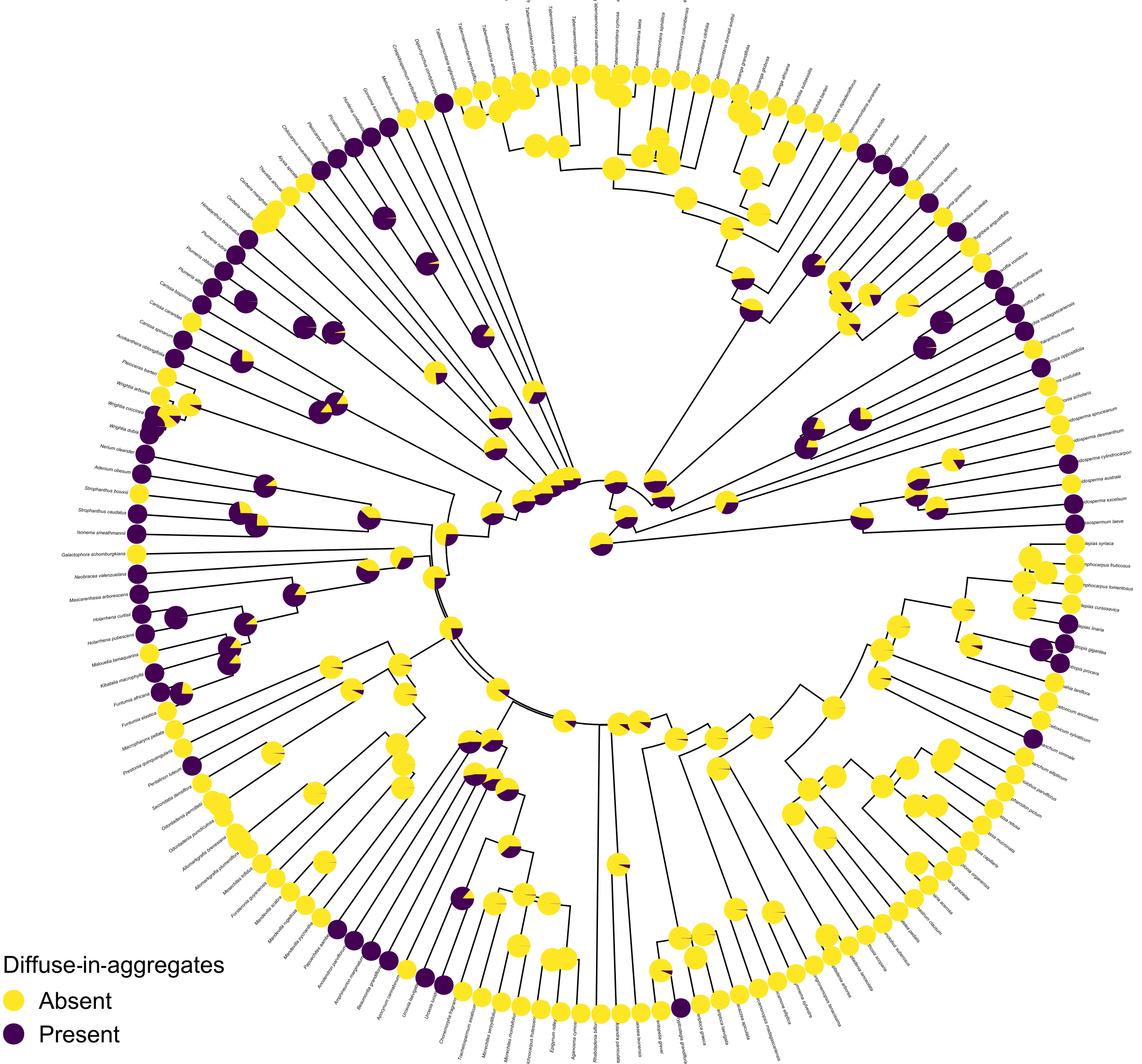
Diffuse-in-aggregates
Absent
Present

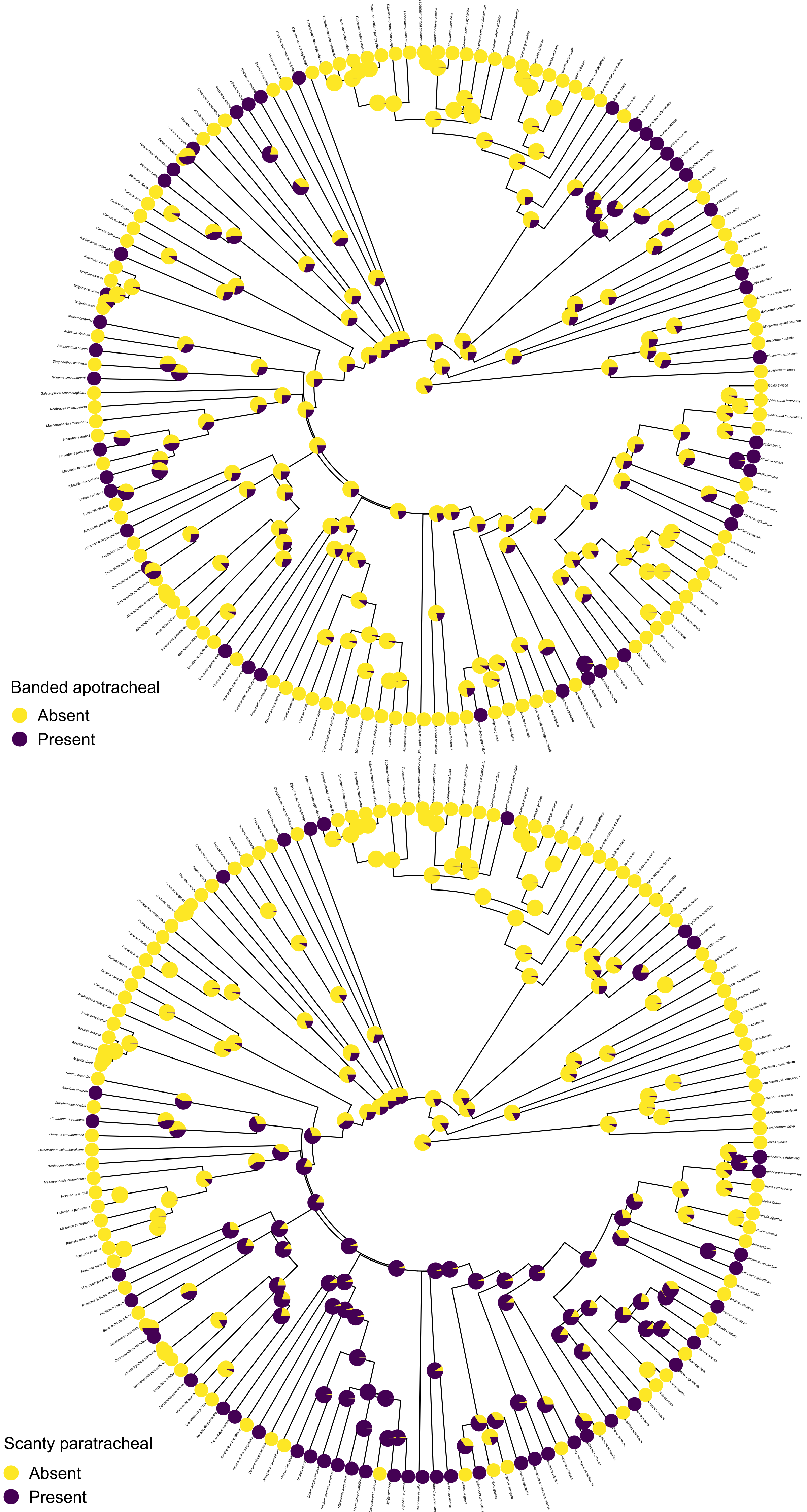
Banded apotracheal
Absent
Present
Scanty paratracheal
Absent
Present

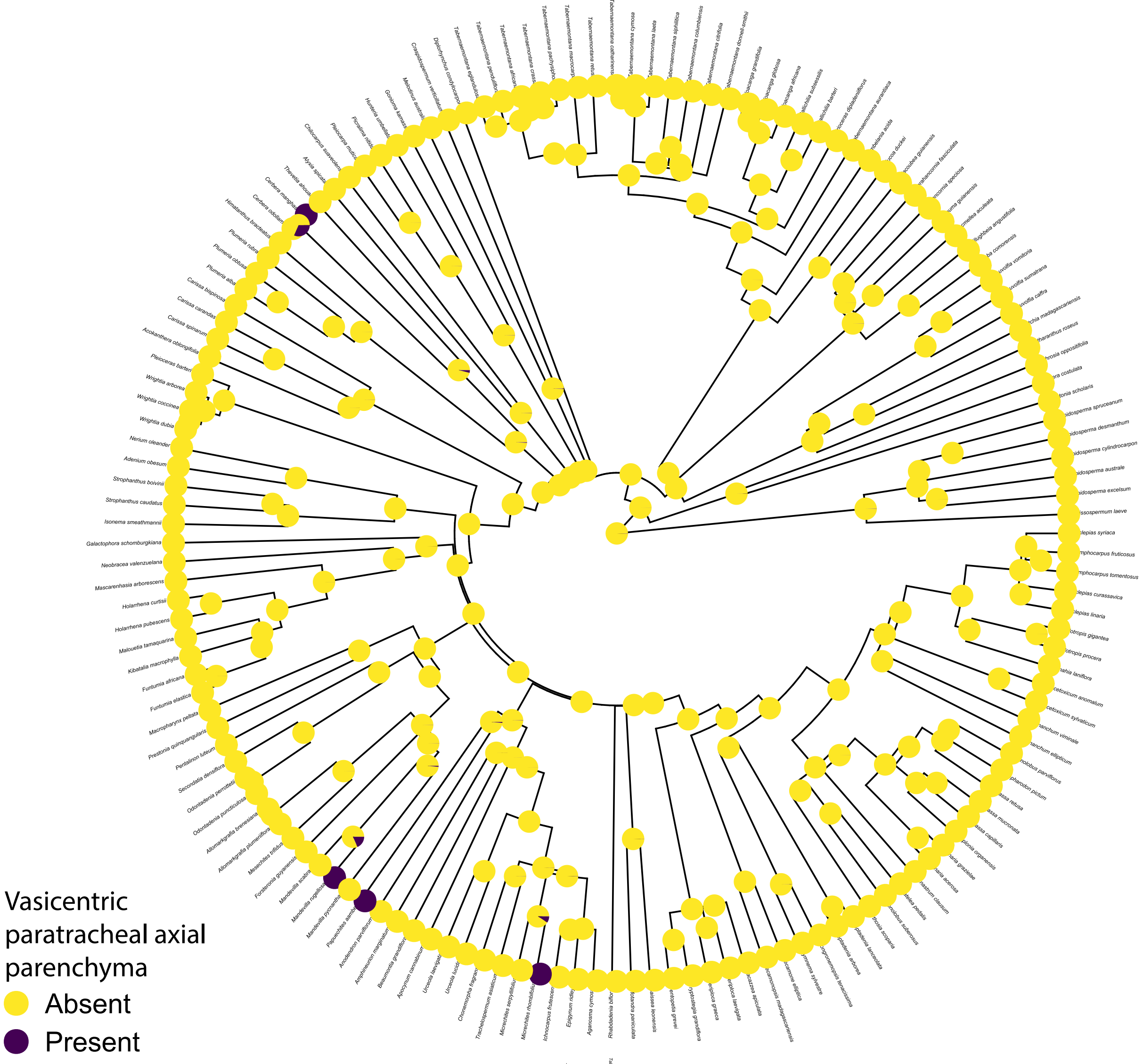
Vasicentric paratracheal axial parenchyma
Absent
Present

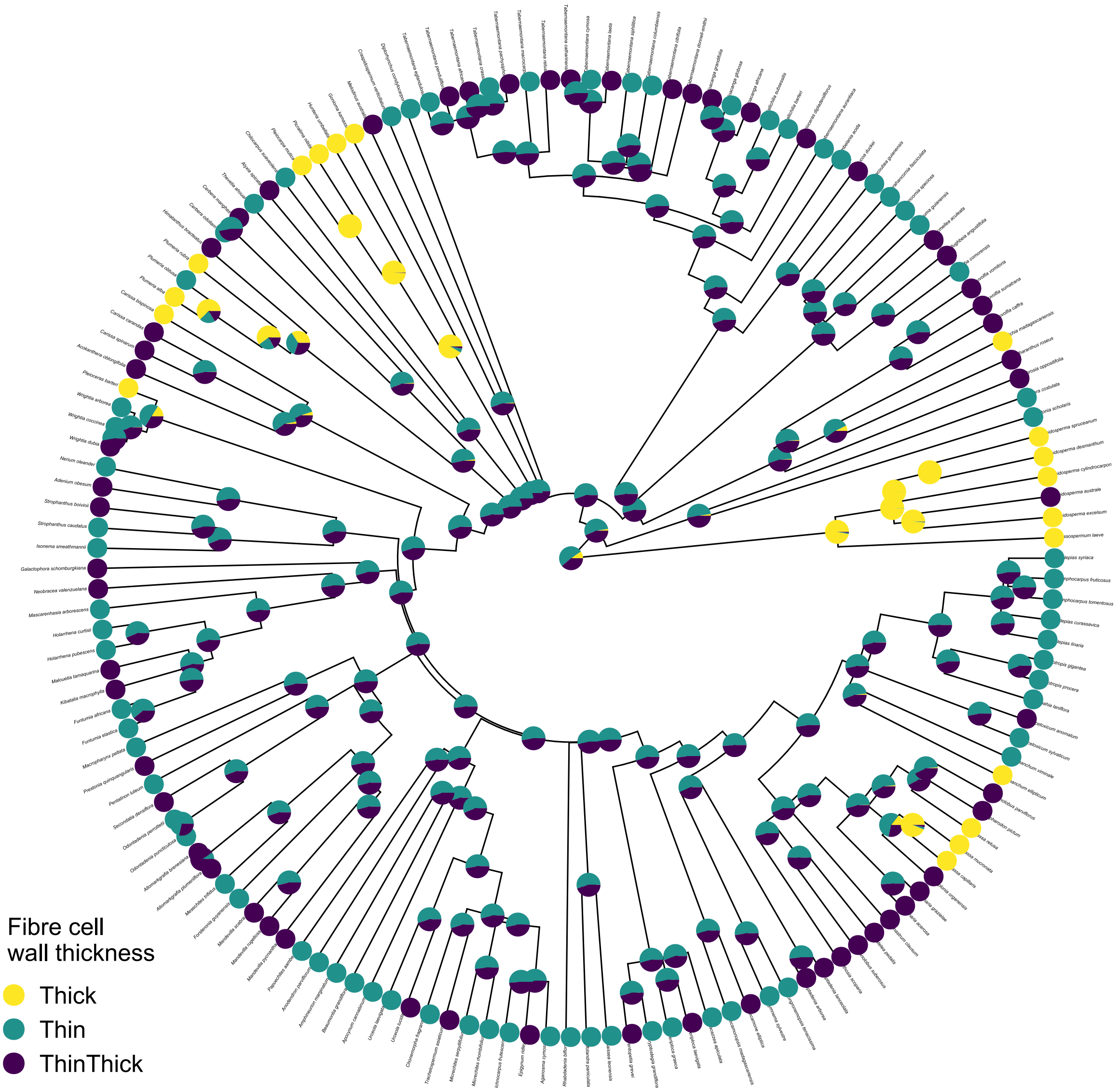
Fibre cell wall thickness
Thick
Thin
ThinThick

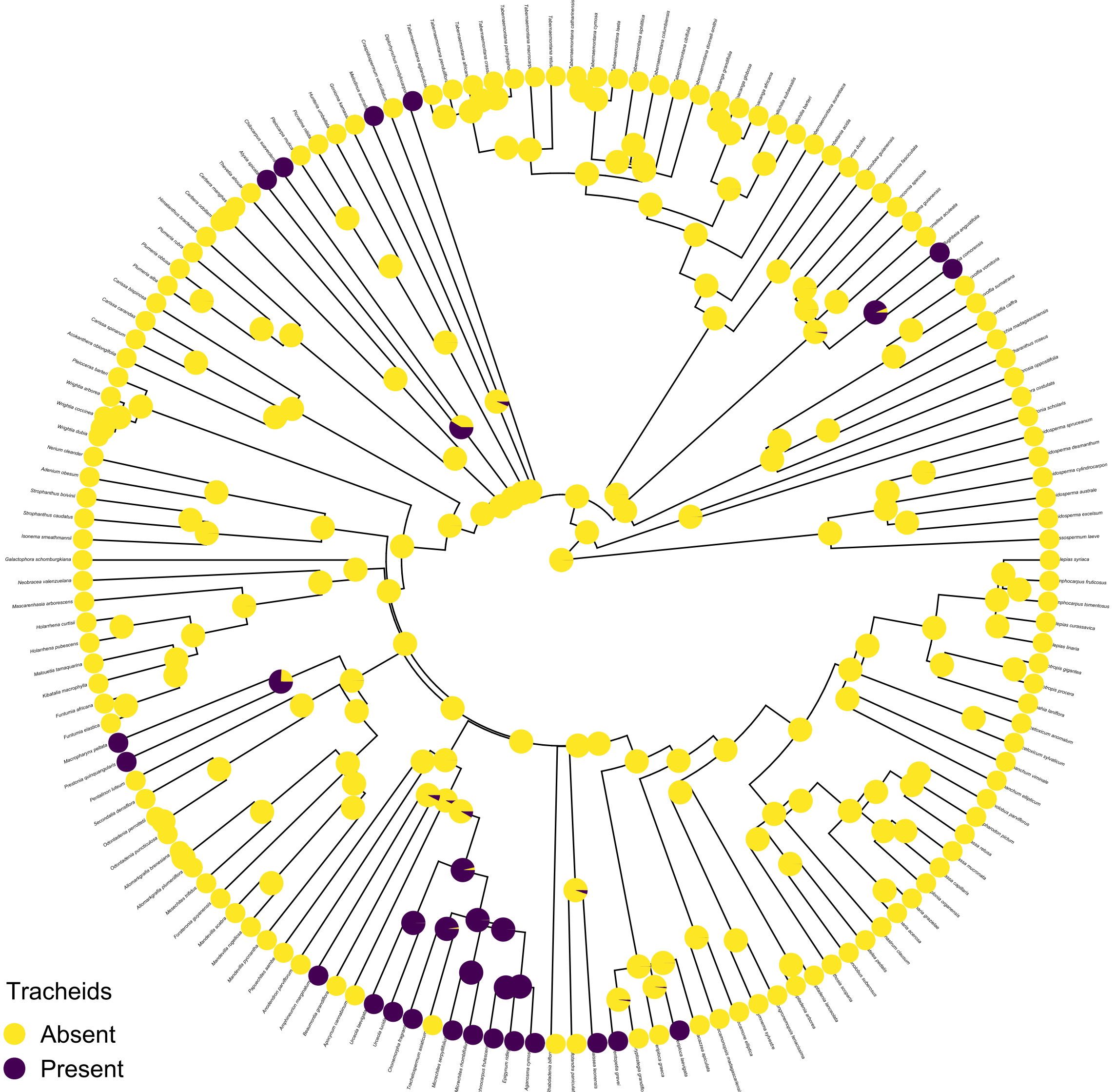
Tracheids
Absent
Present

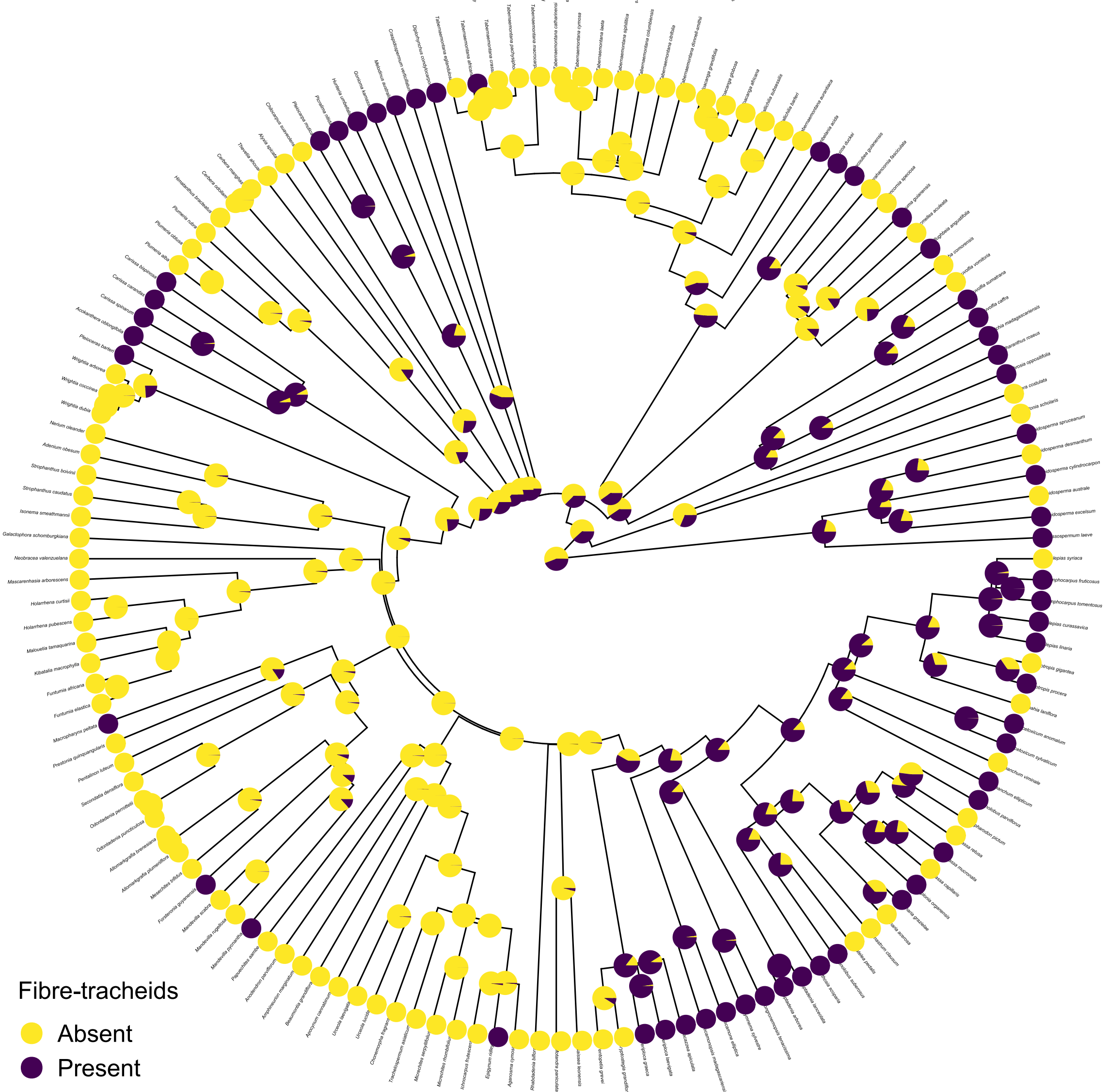
Fibre-tracheids
Absent
Present

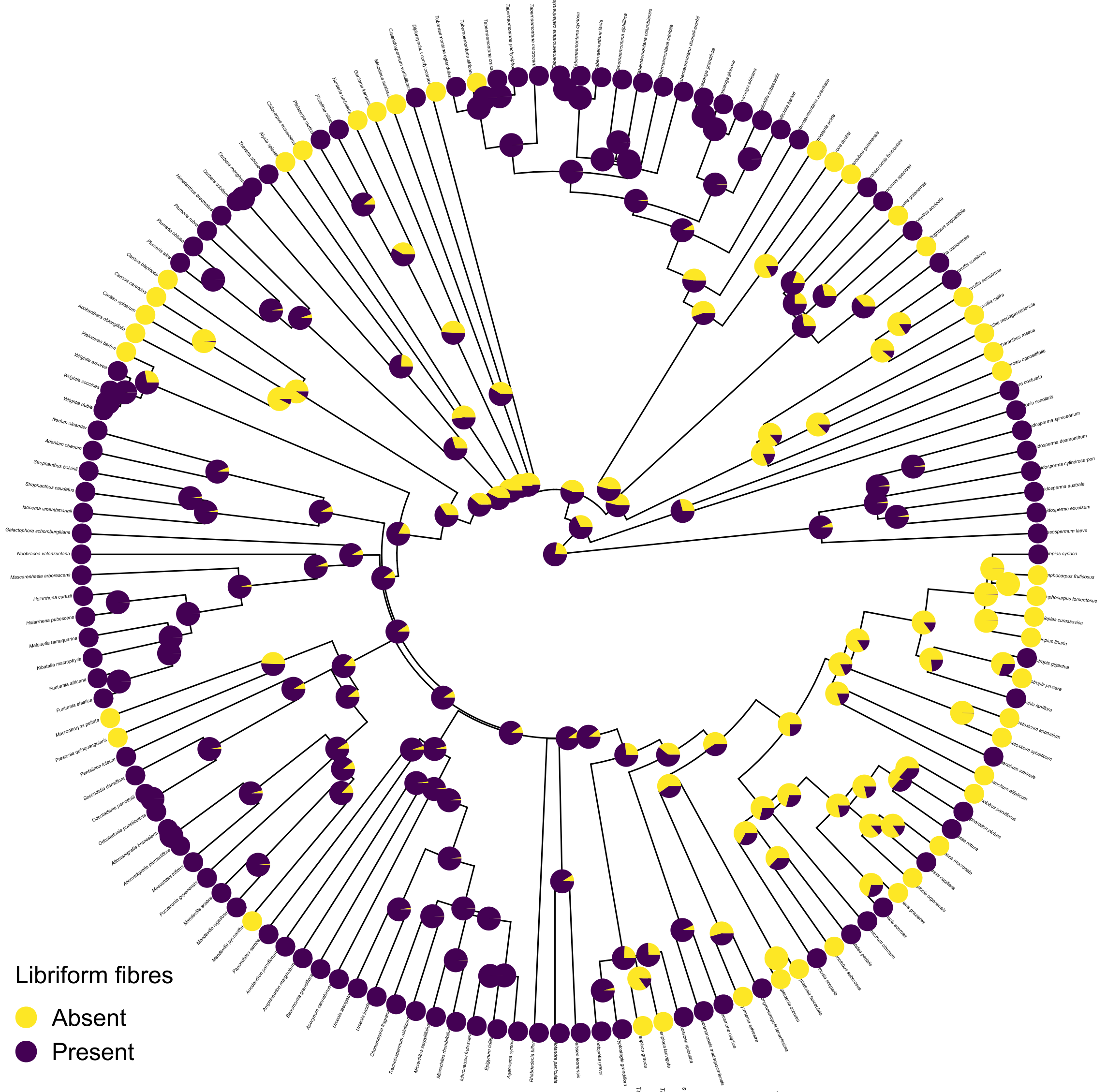
Libriform fibres
Absent
Present

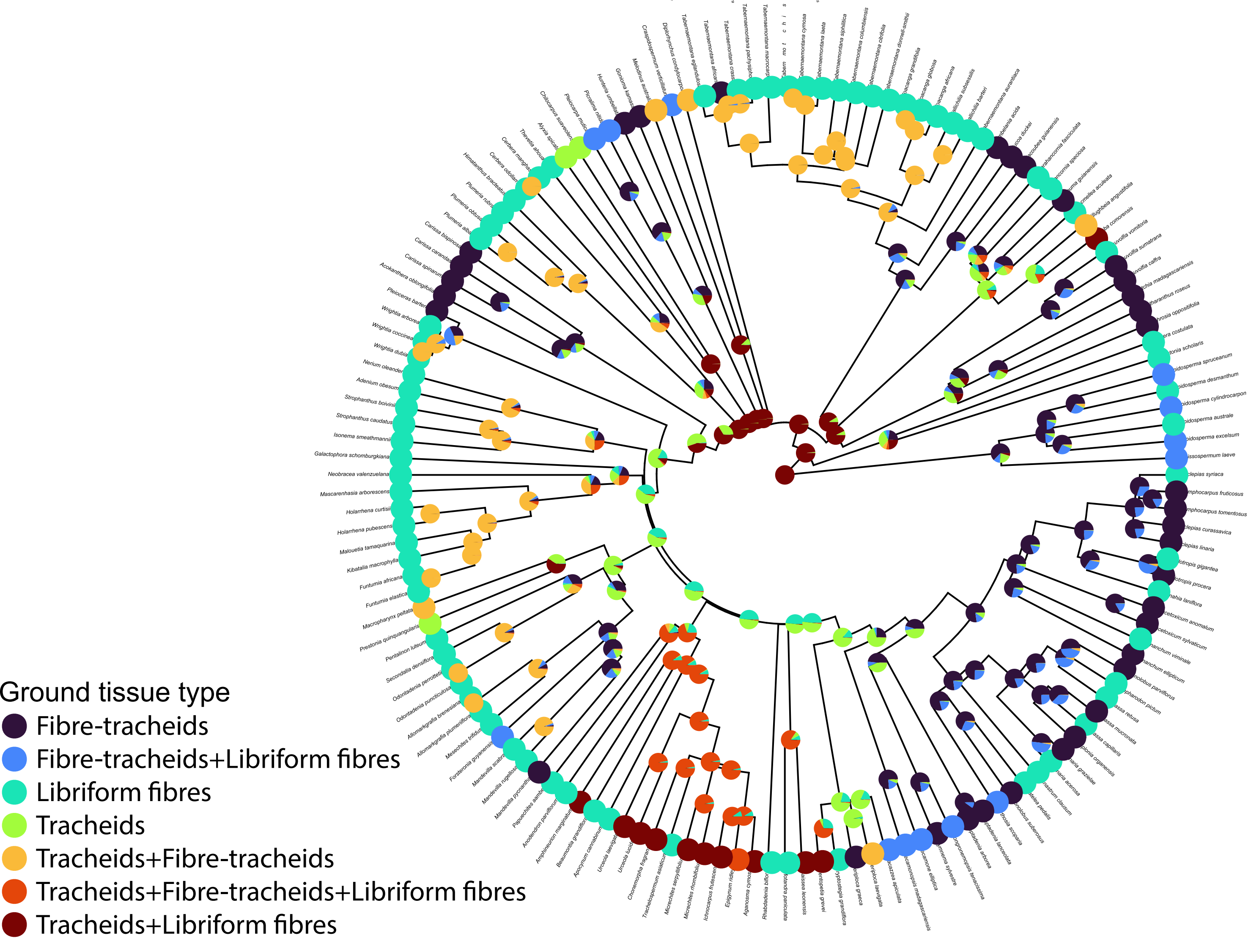
Ground tissue type
Fibre-tracheids
Fibre-tracheids+Libriform fibres
Libriform fibres
Tracheids
Tracheids+Fibre-tracheids
Tracheids+Fibre-tracheids+Libriform fibres
Tracheids+Libriform fibres

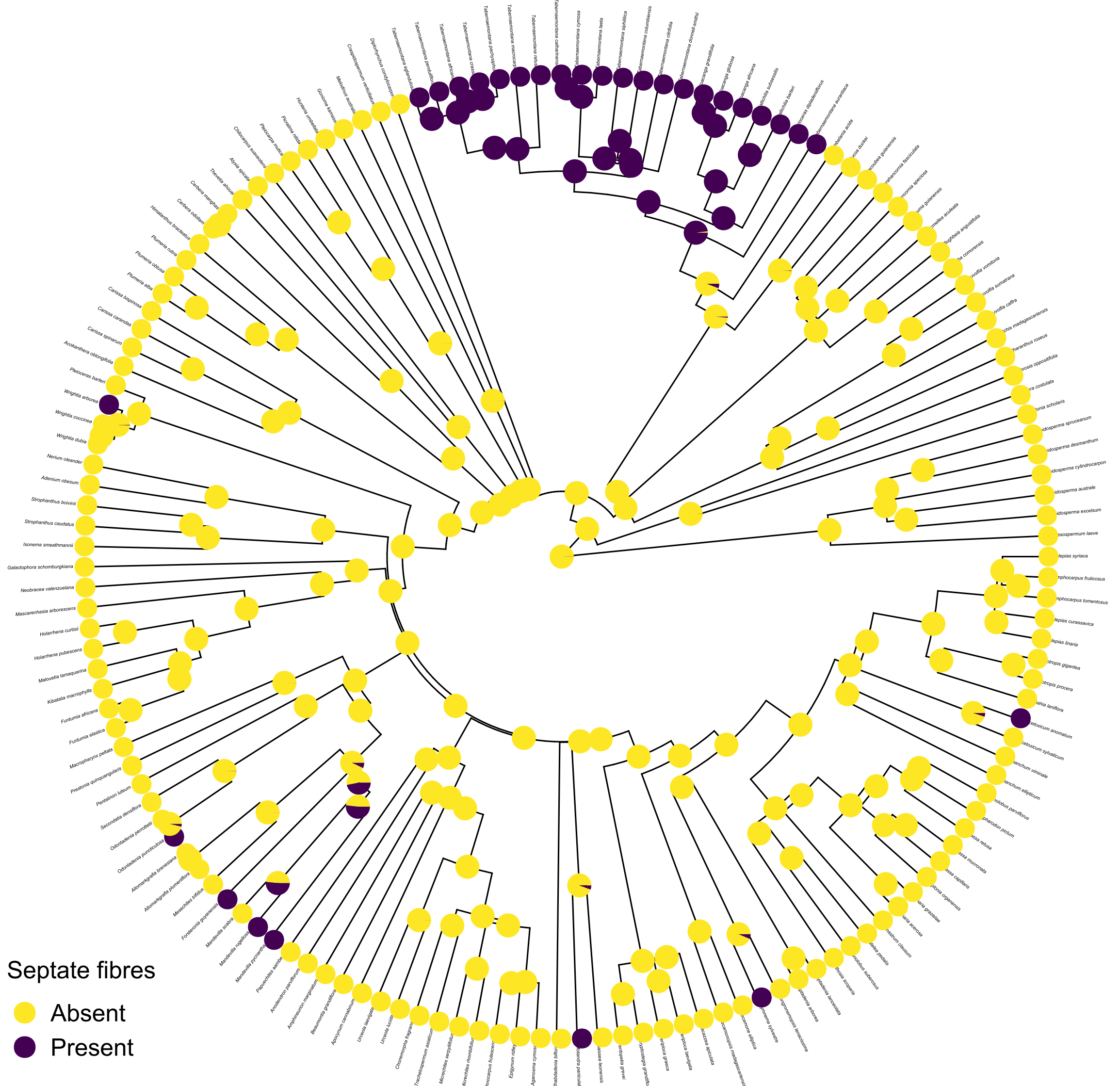
Septate fibres
Absent
Present

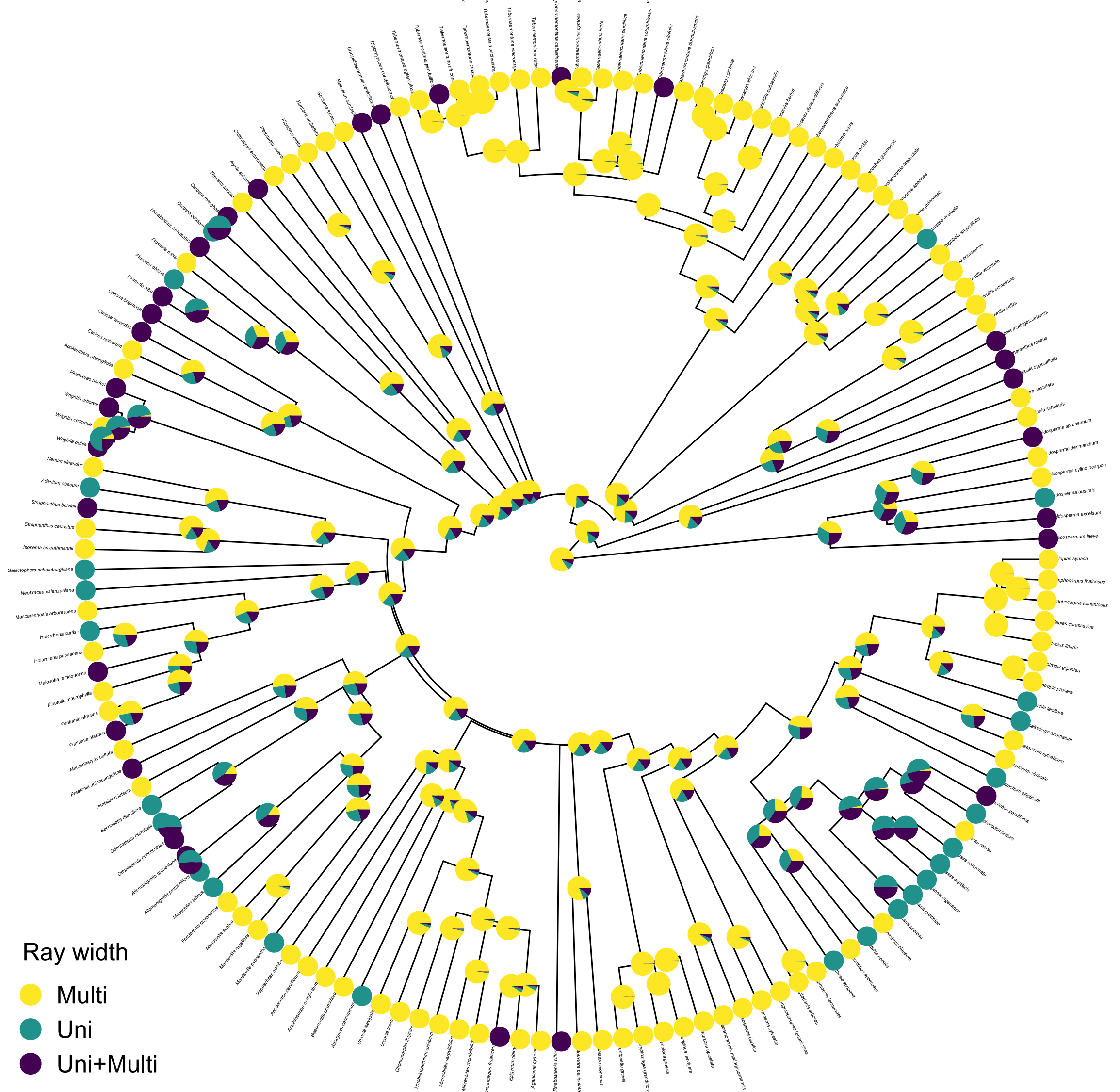
Ray width
Multi
Uni
Uni+Multi

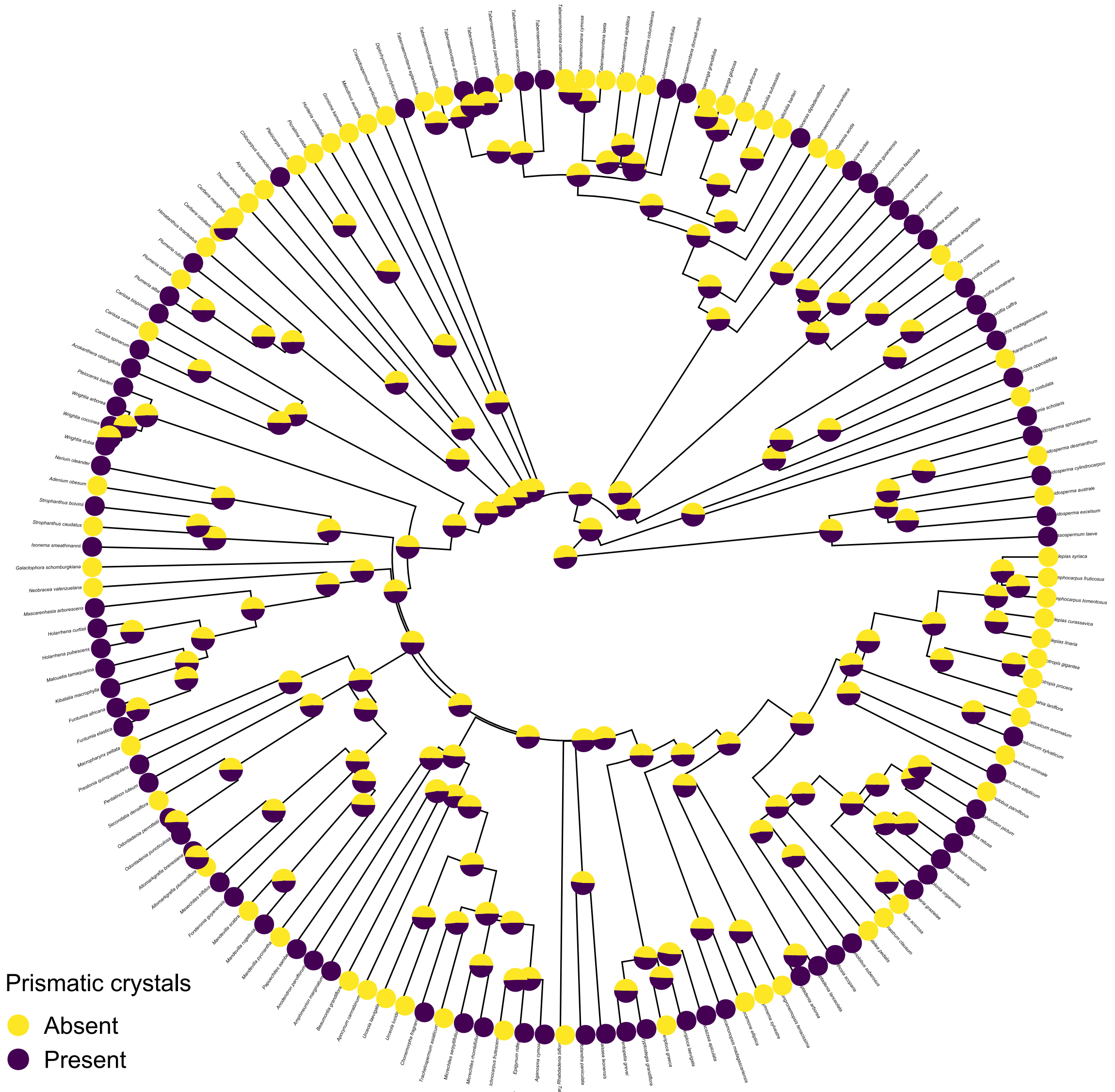
Prismatic crystals
Absent
Present

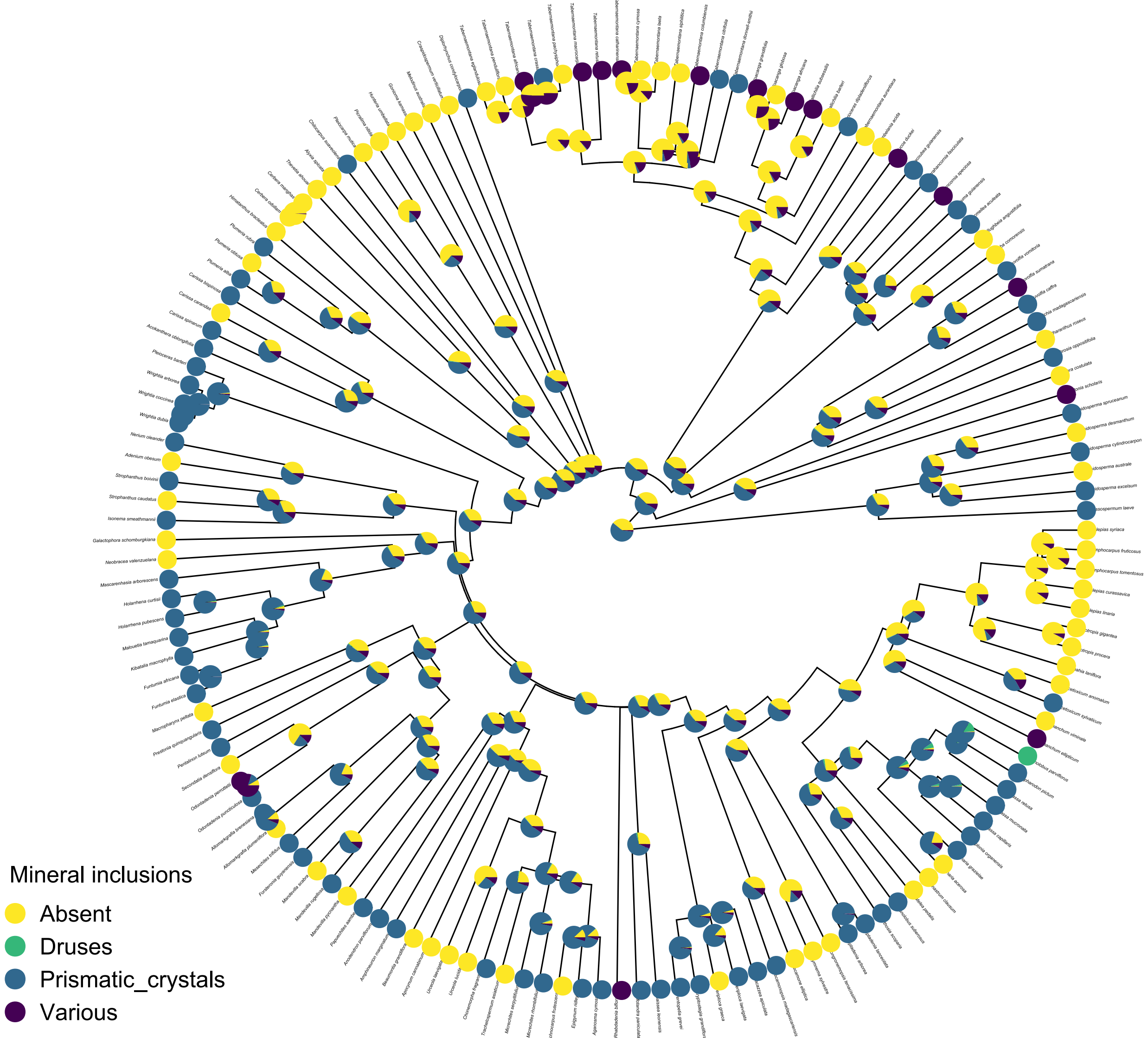
Mineral inclusions
Absent
Druses
Prismatic_crystals
Various

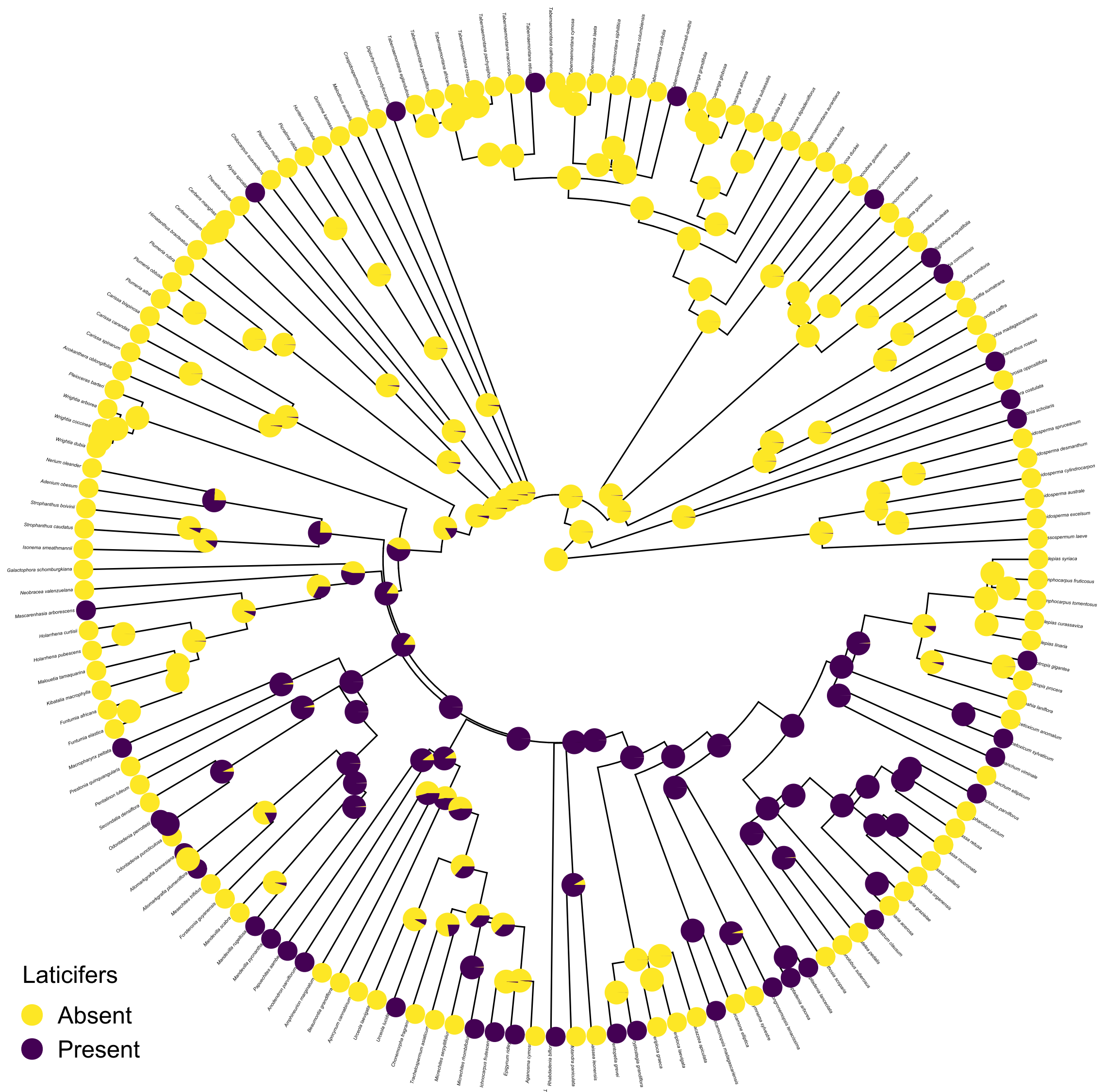
Laticifers
Absent
Present

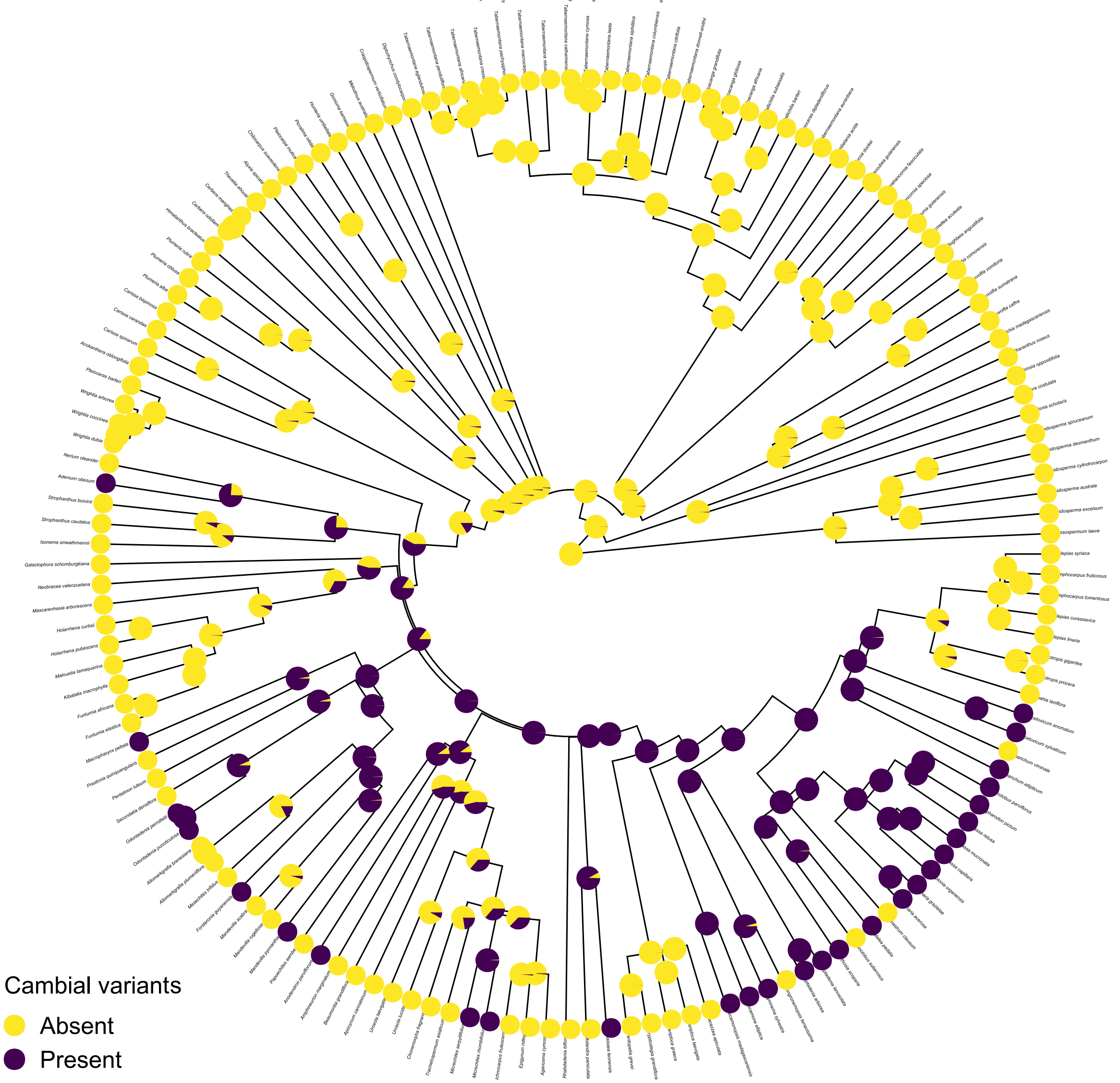
Cambial variants
Absent
Present
